# Linking land-use and land-cover transitions to their ecological impact in the Amazon

**DOI:** 10.1073/pnas.2202310119

**Published:** 2022-06-27

**Authors:** Cássio Alencar Nunes, Erika Berenguer, Filipe França, Joice Ferreira, Alexander C. Lees, Julio Louzada, Emma J. Sayer, Ricardo Solar, Charlotte C. Smith, Luiz E. O. C. Aragão, Danielle de Lima Braga, Plinio Barbosa de Camargo, Carlos Eduardo Pellegrino Cerri, Raimundo Cosme de Oliveira, Mariana Durigan, Nárgila Moura, Victor Hugo Fonseca Oliveira, Carla Ribas, Fernando Vaz-de-Mello, Ima Vieira, Ronald Zanetti, Jos Barlow

**Affiliations:** ^a^Departamento de Ecologia e Conservação, Universidade Federal de Lavras, Lavras, Minas Gerais, 37200-900, Brazil;; ^b^Lancaster Environment Centre, Lancaster University, Lancaster, Lancashire, LA1 4YQ, United Kingdom;; ^c^Environmental Change Institute, University of Oxford, Oxford, OX1 3QY, United Kingdom;; ^d^School of Biological Sciences, University of Bristol, Bristol, BS8 1TQ, United Kingdom;; ^e^Embrapa Amazônia Oriental, Belém, 66095-100, Brazil;; ^f^Department of Natural Sciences, Manchester Metropolitan University, Manchester, M1 5GD, United Kingdom;; ^g^Cornell Lab of Ornithology, Cornell University, Ithaca, NY 14850;; ^h^Smithsonian Tropical Research Institute, Apartado 0843-03092, Balboa, Republic of Panama;; ^i^Departamento de Genética, Ecologia e Evolução, Instituto de Ciências Biológicas, Universidade Federal de Minas Gerais, Belo Horizonte, Minas Gerais, 31270-901, Brazil;; ^j^Earth Observation and Geoinformatics Division, National Institute for Space Research, São José dos Campos, 12227-010, Brazil;; ^k^College of Life and Environmental Sciences, University of Exeter, Exeter, EX4 4RJ, United Kingdom;; ^l^Centro de Energia Nuclear na Agricultura, Universidade de São Paulo, Piracicaba, 13416-000, Brazil;; ^m^Departamento de Ciência do Solo, Escola Superior de Agricultura Luiz de Queiroz, Universidade de São Paulo, Piracicaba, 13418-900, Brazil;; ^n^Museu Paraense Emílio Goeldi, Belém, PA, 66040-170, Brazil;; ^o^Departamento de Biologia e Zoologia, Instituto de Biociências, Universidade Federal de Mato Grosso, Cuiabá, Mato Grosso, 78060-900, Brazil;; ^p^Departamento de Entomologia, Universidade Federal de Lavras, Lavras, Minas Gerais, 37200-900, Brazil

**Keywords:** biodiversity, carbon, deforestation, degradation, logging

## Abstract

Human activities pose a major threat to tropical forest biodiversity and ecosystem services. Although the impacts of deforestation are well studied, multiple land-use and land-cover transitions (LULCTs) occur in tropical landscapes, and we do not know how LULCTs differ in their rates or impacts on key ecosystem components. Here, we quantified the impacts of 18 LULCTs on three ecosystem components (biodiversity, carbon, and soil), based on 18 variables collected from 310 sites in the Brazilian Amazon. Across all LULCTs, biodiversity was the most affected ecosystem component, followed by carbon stocks, but the magnitude of change differed widely among LULCTs and individual variables. Forest clearance for pasture was the most prevalent and high-impact transition, but we also identified other LULCTs with high impact but lower prevalence (e.g., forest to agriculture). Our study demonstrates the importance of considering multiple ecosystem components and LULCTs to understand the consequences of human activities in tropical landscapes.

## Significance

Tropical forests are threatened by human activities, which result in deforestation and degradation. However, multiple land-use and land-cover transitions are occurring in tropical landscapes, and we do not know how these transitions differ in terms of their rates and impacts on the ecosystem. We show that deforestation for pasture was the most prevalent and high-impact transition in the Brazilian Amazon, although other less prevalent transitions also caused a reduction in biodiversity and carbon stocks and altered soil properties. Of all the ecosystem properties we studied, biodiversity was the most affected by all land-use and land-cover transitions. We show the importance of considering the multiple transitions and ecosystem properties to understand the current state and future of tropical forest landscapes.

Tropical forests host two-thirds of all terrestrial biodiversity (1), account for one-third of terrestrial productivity and evapotranspiration (2), and store half of all terrestrial carbon (3, 4). Despite their global importance, tropical forests are being severely affected by human activities (5). Deforestation is a key driver of change—more than 100 million ha of primary tropical forests have been converted to agriculture and silviculture in the last 40 y (6, 7). Many of the remaining primary forests are also degraded. Between 2000 and 2005 at least 20% of tropical forests were selective logged (8), while other anthropogenic drivers and extreme droughts are increasing forest fires, with 54 million ha burned annually in the 1990s (9). Even deforested landscapes are changing; agricultural abandonment is a key driver of secondary forest regrowth, and now these forests account for at least half of all tropical forests globally (10), including 28% of deforested land in the Amazon (11). Other areas are undergoing agricultural intensification, with pastures being converted to croplands (12, 13). Hence, many tropical landscapes are now a mosaic of nonforested land uses, regenerating secondary forests, and variably degraded primary forests (14).

Several studies have quantified the different land-use and land-cover transitions (LULCTs) in tropical forest regions, including in the Amazon (e.g., refs. 15 and 16). We know, for example, that conversion of forests to pastures and degradation of primary forests are the main LULCTs in the Amazon (15, 16), while secondary forest recovery is still very limited compared with deforestation (11). In addition, the value of human-modified tropical landscapes has been examined in detail for above-ground carbon storage (17), soil condition (18), vegetation structure (19), and biodiversity (20). The ecological literature shows that conversion transitions, for example deforestation, are expected to cause the greatest impact on forest ecosystems (21), but degradation of primary forests can also be as harmful as deforestation for biodiversity when it occurs at scale (22). Other studies discuss how fast biodiversity and carbon can recover in regenerating secondary forests, suggesting, for example, that up to 80% of primary forest tree species could be present in 40-y-old Amazonian secondary forests (23), while a meta-analysis suggests that, even after a century, plant species diversity does not recover to undisturbed tropical forest levels (24).

Although the scientific community has been building a solid knowledge base on LULCTs in tropical forest regions, the existing studies do not reveal the full extent of changes in landscape condition for four key reasons: First, a compilation of the rates of different types of LULCTs, including deforestation, regeneration, and forest degradation, is still lacking, impeding a comprehensive view of LULCT dynamics that allows a quantitative comparison between all transitions. Second, region-specific studies often focus on one or two ecosystem components (24–26) or compare changes with an undisturbed forest baseline rather than evaluating the full range of transitions (23, 27). Third, meta-analyses have focused on 1) a single ecosystem component (18), 2) a single type of LULCT (28), or 3) comparing all changes with an undisturbed baseline, without exploring the transitions that occur between human-modified land uses (21). Finally, ecological changes have not been linked to prevalence (e.g., measured as annual rates) of the land-cover transitions to date. Quantifying transitions in terms of prevalence and impact on ecosystem properties is a key step toward understanding the relative importance of changes across whole landscapes and could provide evidence-based scenarios for policymakers to decide how to better protect and benefit from tropical forest biodiversity and ecosystem services.

Here, we quantified the prevalence and the ecological impacts of 18 LULCTs in the Brazilian Amazon to make a comprehensive assessment of the relative risk of the major LULCTs. We focused on the following questions: 1) What are the rates of LULCTs? 2) What are the impacts of LULCTs on different ecosystem properties (e.g., biodiversity, carbon, and soil)? 3) Which transitions impose the greatest magnitude of change on the ecosystem? Ecological data encompassed 18 variables sampled at 310 sites in two distinct regions of the Brazilian Amazon, which we grouped into three ecological dimensions that reflect policy or management levers: biodiversity (vascular plants, birds, and three invertebrate groups), carbon pools, and soil properties. These ecosystem components were surveyed in seven land-use or land-cover classes: undisturbed primary forest, logged primary forest, logged-and-burned primary forest, old secondary forest (>20 y since abandonment), young secondary forest (≤20 y old), pasture, and mechanized agriculture. The impacts of 18 LULCTs on ecosystem variables were then assessed against the annual LULCT rates estimated between 2006 and 2014 (29) or 2006 and 2019 (15), providing an understanding of both the magnitude and extent of changes in land use and land cover in the Brazilian Amazon.

## Results

### Rates of LULCTs.

**Fig. 1.**
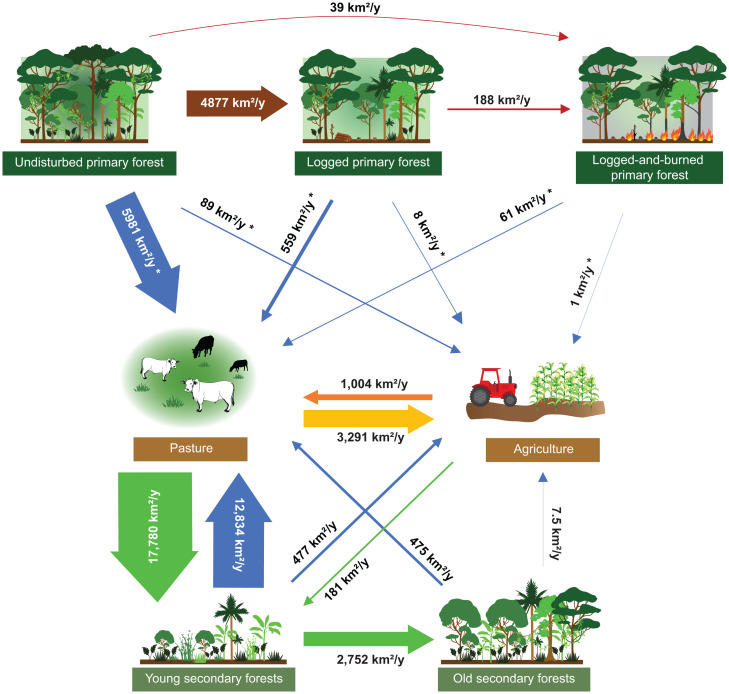
LULCT rates in the Brazilian Amazon. Mean annual LULCT rates were calculated and estimated (*) based on land-use change maps [2006 to 2019 (15)] and on forest degradation maps [2006 to 2014 (29)]. Primary forests have never been clear-cut, and secondary forests are regenerating forests. Young secondary forests are <20 y old and old secondary forests are ≥20 y old. Agriculture includes perennial and temporary crops. The width of the arrows is proportional to the mean annual rate of the transition.

Based on land-use change maps for the period 2006 to 2019 (15, 30) and forest degradation maps for the period 2006 to 2014 (29), the highest rates of LULCTs were from pastures to young secondary forests (17,780 ± 2,840 km^2^⋅y^−1^) followed by transitions from young secondary forests and undisturbed primary forests to pastures (12,834 ± 1,657 and 5,981 ± 2,711 km^2^⋅y^−1^, respectively; Fig. 1). In addition, 4,877 ± 1,218 km^2^⋅y^−1^ of undisturbed primary forests were logged between 2006 and 2014 (Fig. 1). Two other transitions exceeded 2,000 km^2^⋅y^−1^: young secondary to old secondary forests (2,752 ± 536 km^2^⋅y^−1^) and pastures to agriculture (3,291 ± 1,263 km^2^⋅y^−1^; Fig. 1). Indeed, the conversion rate of pastures to agriculture was three times higher than the inverse (agriculture to pasture: 1,004 ± 286 km^2^⋅y^−1^). The conversion rates of primary forests and young and old secondary forests to agriculture were, respectively, 67, 26, and 63 times lower than the conversions to pastures (Fig. 1 and *SI Appendix*, Fig. S2*A*).

### Impacts of LULCTs on Biodiversity, Carbon, and Soil.

**Fig. 2.**
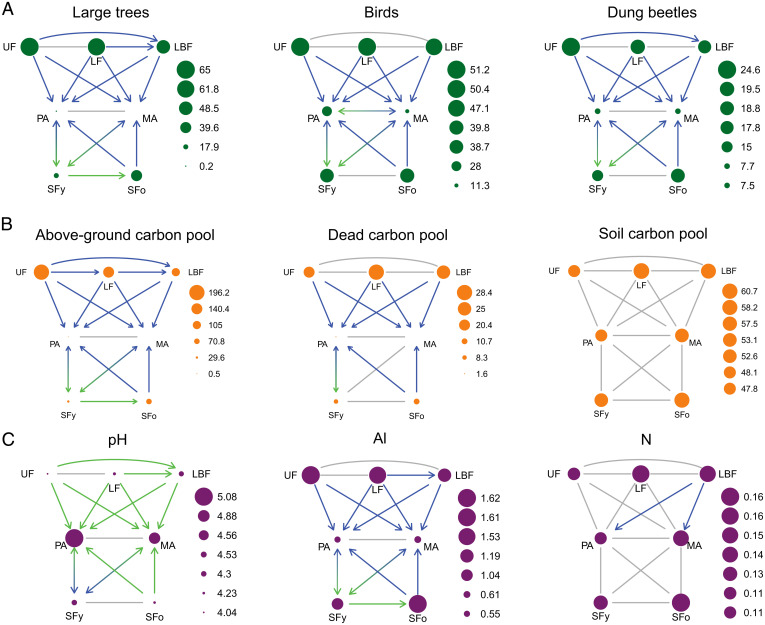
Impacts of 18 LULCTs on (*A*) biodiversity, (*B*) carbon pools, and (*C*) soil properties in the Brazilian Amazon. Biodiversity is given as species richness, carbon pools are given as Mg⋅ha^−1^ of carbon, the unit for Al (aluminum) is mmolc⋅dm^−3^, and N (nitrogen) is given in % soil mass. Arrows indicate the transitions and their effect, where gray indicates no effect, green is a significant increase, and blue is a significant decrease. The size of symbols represents averages based on 21 undisturbed primary forests (UF), 68 logged primary forests (LF), 65 logged-and-burned primary forests (LBF), 72 pastures (PA), 26 mechanized agriculture fields (MA), 33 young secondary forests (SFy, <20 y old), and 25 old secondary forests (SFo, ≥20 y old) distributed in two regions of the eastern Brazilian Amazon.

The species richness (i.e., taxonomic diversity, henceforth diversity) of almost all biodiversity groups declined by 18 to 100% with the conversion of primary and secondary forests to pastures or mechanized agriculture (see *SI Appendix*, Tables S3 and S4 for mean values and % of change for each variable, respectively). Notable exceptions were the diversity of ants and orchid bees, which did not change after the conversion of undisturbed primary forests and old secondary forests to pastures, and the diversity of orchid bees, which did not change after the conversion of any primary forests or old secondary forests to mechanized agriculture (Fig. 2*A* and *SI Appendix*, Fig. S2). Large tree and dung beetle diversity decreased by 25 and 27%, respectively, in response to the transition from undisturbed to logged-and-burned primary forests. The diversity of large trees, small trees, and lianas declined (by 21, 17, and 21%, respectively) when logged primary forests transitioned to logged-and-burned primary forests, and the diversity of ants and birds decreased by 30 and 59%, respectively, when pastures were converted to mechanized agriculture (Fig. 2*A* and *SI Appendix*, Fig. S2 and Table S4). Finally, the diversity of large trees doubled and small tree diversity increased by 55% in response to the transition from young to old secondary forests (Fig. 2*A* and *SI Appendix*, Fig. S2). Separate analysis of forest species produced results that largely mirrored the patterns for total species richness (*SI Appendix*, Figs. S3 and S4).

The above-ground, litter, and dead wood carbon pools decreased by 74 to 100% when primary or secondary forests were converted to pastures or mechanized agriculture (Fig. 2*B* and *SI Appendix*, Fig. S5 and Table S4). The above-ground carbon pool decreased by 28 to 46% in response to the transition from undisturbed to logged or logged-and-burned primary forests, and by 25% in response to the transition from logged to logged-and-burned primary forests (Fig. 2*B*). Only the above-ground carbon pool increased in response to the transition from young to old secondary forests (by 2.4 times; Fig. 2*B* and *SI Appendix*, Fig. S5). The soil carbon pool did not differ among any of the seven land-use and land-cover classes.

Of the seven soil properties assessed, there was an increase between 6% and 4.2 times in soil pH and phosphorus, potassium, and calcium + magnesium concentrations in response to most of the transitions from primary or secondary forests to pasture and mechanized agriculture, and between 6% and 2.8 times from undisturbed to logged or logged-and-burned primary forests (Fig. 2*C* and *SI Appendix*, Fig. S6 and Table S4). Soil pH increased by 5% and calcium + magnesium increased by 50% with the transition from logged to logged-and-burned primary forests. By contrast, soil aluminum concentrations decreased by 41 to 66% when primary or secondary forests were converted to pasture or agriculture (Fig. 2*C*) but increased by 54% in response to the transition from young to old secondary forests (Fig. 2*C*). Soil potassium increased by 25% with the transition from pasture to mechanized agriculture (Fig. 2*C* and *SI Appendix*, Fig. S6). Soil nitrogen percentages only changed in response to the conversion of logged-and-burned forests to pasture and mechanized agriculture (Fig. 2*C*). Finally, soil sodium concentrations did not differ among any of the seven land-use and land-cover classes (*SI Appendix*, Fig. S6).

### Magnitude of Ecosystem Change with LULCTs.

**Fig. 3.**
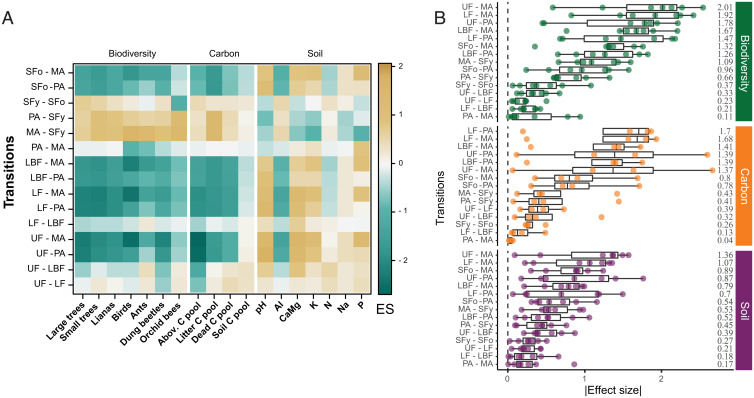
Standardized effect sizes of LULCTs on the ecosystem in the Brazilian Amazon. (*A*) Standardized effect sizes (ES) of 15 LULCTs (three of which are bidirectional and are shown only once: PA–MA, PA–SFy, and MA–SFy) on seven biodiversity groups, four carbon pools, and seven soil properties. (*B*) Boxplots of the absolute values of effect sizes (dots) for the impact of 15 LULCTs on biodiversity, carbon pools, and soil properties in the Brazilian Amazon. The numbers in *B*, *Right* are the median standardized effect sizes, converted to absolute values. The standardized effect sizes were derived from mixed-effects models analyzing the impact of LULCTs on individual variables within an ecosystem component. Note that in *B* the transitions are ordered from largest to smallest impact based on their median effect sizes and therefore differ among ecosystem components. The transitions MA–SFy, PA–SFy, and PA–MA are bidirectional.

Based on the ranking of transitions by their median effect size, the greatest declines in biodiversity occurred in the transitions from primary and secondary forest classes to mechanized agriculture, followed by the transitions from the forest classes to pasture (Fig. 3*B*). The bidirectional transition between pasture and mechanized agriculture had the smallest impact on biodiversity, although the individual impact on bird and ant diversity was intermediate (Fig. 3). The next-smallest magnitudes of change for biodiversity were observed in the transitions among primary forest classes 1) from undisturbed to logged or logged-and-burned primary forests, and 2) between logged and logged-and-burned primary forests (Fig. 3*B*). Similarly, the transitions from primary forest classes and old secondary forests to pasture or mechanized agriculture had the highest impacts on carbon pools and soil properties, followed by transitions between pasture and mechanized agriculture to young secondary forests (Fig. 3). The transition between pasture and mechanized agriculture had the lowest impact on carbon pools and soil properties, followed by the transitions between primary forest classes and from young to old secondary forests (Fig. 3). The transition from undisturbed primary forest to mechanized agriculture had the greatest impact on biodiversity and soil ecosystem components (Fig. 3), while the two highest individual effect sizes were recorded for the above-ground carbon pool in response to undisturbed primary forest conversion to mechanized agriculture and pasture (Fig. 3).

### Linking Impacts with Transition Rates.

**Fig. 4.**
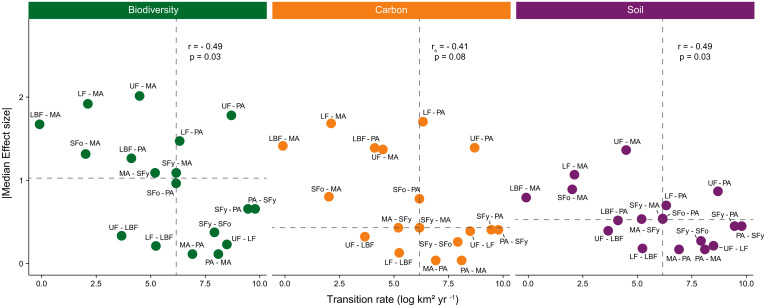
Relationship between LULCT rates and their impacts on ecosystem components in the Brazilian Amazon. Within each ecosystem component (biodiversity, carbon, or soil), the dotted lines represent medians separating four classes of transitions: high impact, low rate (*Top Left*), high impact, high rate (*Top Right*), low impact, high rate (*Bottom Right*), and low impact, low rate (*Bottom Left*). Mean annual rates (km^2^⋅y^−1^) are log-transformed, and the impacts are represented by the median standardized effect sizes (converted to absolute values) obtained from models assessing the effects of 18 LULCTs on seven biodiversity groups, four carbon pools, and seven soil properties.

The rate at which LULCTs occurred was inversely correlated to their impacts on biodiversity (*r* = −0.49, *P* = 0.03) and soil properties (*r* = −0.49, *P* = 0.03; Fig. 4), but we found no correlation between these variables for the carbon ecosystem component (*P* = 0.08; Fig. 4). However, using median annual transition rates and effect sizes to class the rates and impacts of a transition for each ecosystem component, we found that transitions from undisturbed or logged primary forests to pasture were always classed as high-impact, high-rate regardless of the ecosystem component. Most of the transitions to mechanized agriculture were classed as high-impact, low-rate, as these transitions often had the greatest impact but occurred at lower annual rates (e.g., conversion of undisturbed primary forests). The bidirectional transition between pasture and mechanized agriculture and the transitions from young secondary forests to old secondary forests and from young secondary forests to pastures were all classed as low-impact, high-rate for the three ecosystem components. Although the transition from undisturbed primary forests to logged forests occurred at high annual rates, the median effect sizes for all ecosystem components were low, and the transition was therefore also classed as low-impact, high-rate. Finally, the transitions from undisturbed and logged primary forests to logged-and-burned primary forests were classed as low-impact, low-rate (Fig. 4).

## Discussion

Our study examines the prevalence and impacts of LULCTs on tropical ecosystems. We identified a negative relationship between LULCT rates and impacts on biodiversity and soil properties, which suggests that the greatest impacts are affecting smaller areas of the Brazilian Amazon. However, by identifying the relative risk of LULCTs to ecosystem components, we also highlighted transitions that were both high-impact and prevalent, as well as those that had a high impact but occurred less frequently. By doing that, we provide a methodology that can be applied to provide more specific guidance for landscape-scale conservation or restoration efforts than has hitherto been available. Deforestation for cattle ranching remains the most widespread anthropogenic activity in the Amazon (31, 32), and the transitions from undisturbed or logged primary forests to pastures were the only we classed as high-impact, high-rate. Transitions from primary and secondary forests to pasture amounted to 24,000 km^2^⋅y^−1^ and resulted in the complete loss of the above-ground carbon pool and major losses of bird and invertebrate diversity of between 18 and 69%. These diversity effects also include species replacement, in which forest species are replaced by species from open areas, and hence the magnitude of changes would likely be higher if species composition were taken into account (20, 22). Transitions from primary and secondary forests to mechanized agriculture had the most impact but occurred less frequently. However, mechanized agriculture is only prevalent in some specific regions of the Amazon, where productive systems established for grain production and exportation dominate (33). Although primary forest degradation can be as widespread as deforestation (29, 34), the transitions from undisturbed to logged or logged-and-burned primary forests had a much lower impact, highlighting the importance of protecting disturbed forests from further clearance (e.g., ref. 35). Nonetheless, these transitions reduced the value of the forest for biodiversity, carbon, and soil, showing the relevance of actions that limit degradation as well as deforestation—especially as the cumulative impact of degradation can be as detrimental as deforestation when degradation occurs across large spatial scales (22). Finally, the two most prevalent transitions were from pasture to young secondary forest and the reverse (17,780 and 12,834 km^2^⋅y^−1^, respectively), reflecting the inefficiency of extensive pastures, which perform at only ∼30% of the potential productivity in Brazil (36).

Biodiversity and carbon pools were the most sensitive ecosystem components to many of the transitions. This is concerning, because there are numerous examples of how biodiversity loss and turnover reduce the efficiency and stability of critical ecosystem services (37, 38) and undermine our ability to develop nature-based solutions to both the climate and biodiversity crisis (39). In addition, the negative effects of LULCTs on vegetation not only directly affect carbon stocks, forest structure, and plant diversity but also have cascading impacts on the diversity of many other taxa (40). Orchid bees deserve a specific mention because they were insensitive to most LULCTs. Orchid bees forage long distances and can disperse even in fragmented landscapes (41), making them relatively resistant to land-use and land-cover changes. In addition, they are trapped using a chemical bait that may attract them across very large distances. However, it is also possible that the high level of forest cover in the landscape of both studied regions (>50%) helped to buffer the impacts of LULCTs but lower levels of habitat amount in the landscape could cascade with greater impact (42). In addition, although orchid bee diversity did not respond to many LULCTs, it remains to be seen whether associated pollination services are also unaffected.

Many soil properties—including soil carbon, nitrogen, and sodium—appeared insensitive to deforestation, whereas soil pH, calcium + magnesium, potassium, and phosphorus increased, likely reflecting fertilizer and lime application or inputs from livestock (43, 44). Although increased soil pH and nutrient concentrations in human-modified habitats could potentially facilitate the establishment of competitive fast-growing tree species during regeneration [e.g., in restoration plantings (45)], there is little evidence this is happening at present with higher land-use intensity: the recovery rates of secondary forests tend to be lower on sites previously occupied by pasture and mechanized agriculture (46). In addition, it seems likely that nutrient-rich agricultural systems are not the ones that are currently regenerating to secondary forests because these productive lands are less prone to being abandoned. Furthermore, given the limited current understanding of soil processes in the tropics, it is unclear how some of the observed changes in soil physicochemical properties could cascade through soil microbiota and affect soil functioning, especially in the long term (47).

The conversion of old secondary forests (≥20 y) to pasture or agriculture had similar effects on ecosystem components to the conversion of primary forests. Although secondary forests are not substitutes for undisturbed primary forests, our results reinforce the high potential value of older secondary forests, especially those that have regenerated naturally in regions that retain more than 50% of forest cover (48); such forests have biodiversity (23), carbon stocks (24), and soil conditions that may be similar to primary forests (27). Our findings therefore provide strong empirical support for legislation to protect older secondary forests from clearance [e.g., in the State of Pará (23, 49)], especially in light of the recent acceleration of secondary forest clearance in the Amazon (50). Unfortunately, the low rate of the transition from young to old secondary forests suggests that secondary forests are being cleared within the first 20 y of regrowth. Hence, significant benefits for biodiversity and climate change mitigation could also be accrued from protecting these younger secondary forests and allowing them to mature.

The bidirectional transition between pasture and agriculture was extensive, affecting 4,295 km^2^⋅y^−1^ and the rate has been increasing over time between 2006 and 2019 (*SI Appendix*, Fig. S7). In some regions, the transition from pasture to mechanized agriculture is replacing the transitions from forest to mechanized agriculture that occurred in the early 2000s, because of incentives to avoid further deforestation (36). Although the pasture–mechanized agriculture transition had the lowest impact on biodiversity, carbon, and soil, we only analyzed its impacts on the terrestrial ecosystem. Aquatic ecosystems are likely to be especially susceptible to transitions involving agricultural intensification (51). Phosphorus and nitrogen loads, turbidity, and temperature in streams can all increase in response to agricultural intensification and can lead to a cascade of effects on biodiversity and ecosystem services (51).

Our results highlight the large variation in the responses to different LULCTs within certain ecosystem components (Fig. 3*B*), with particularly marked differences among carbon pools. For example, although the above-ground carbon pool decreased by 99% with the conversion of undisturbed primary forest to pasture, the soil carbon pool remained unchanged. The responses of different biodiversity groups also varied markedly, depending on the LULCT. For example, the diversity of all vegetation groups decreased with the transition from logged to logged-and-burned primary forests, but none of the fauna groups were affected. It is conceivable that colonization of open-habitat species has compensated for the loss of forest species (52), and a stricter definition of forest species (e.g., forest specialists) would likely reveal stronger effects of fires and logging (22). Besides the intrinsic nature of the different variables we included in our study, this high variability can also result from differences in the intensity, frequency, and time since the transition happened, as well as the specific context of the landscape [e.g., the extent of surrounding forest cover (53, 54)].

### Implications for Policy and Practice.

Identifying the most important transitions and sensitive properties of the ecosystem is the first step in developing a fuller understanding of the current state and possible future of tropical forests. We believe our results have five important implications for policy. First, they provide compelling evidence that deforestation of primary forests to create pasture remains the most important high-impact, high-rate land-use transition in the Brazilian Amazon. Thus, we highlight the vital importance of combating deforestation in the Amazon, which reached the highest rate of the decade in 2020 and is likely to increase in the future (55). Second, by linking the prevalence and the impact of LULCTs, our analysis helps to define local and regional immediate and long-term actions, highlighting the transitions that should be prioritized. For instance, limiting the transitions we identified as high-impact, high-rate requires the most immediate efforts at the biome scale, but curbing high-impact, low-rate transitions can also be prioritized at local scales (33). Third, our findings demonstrate that improving ecological condition also requires measures that go beyond simply tracking and mitigating deforestation, as transitions involving secondary regeneration and primary forest degradation influenced a range of biodiversity, carbon, and soil metrics. Integrated landscape management will require improvements in monitoring and reporting land-cover transitions to support new policies that incentivize positive transitions, limit the occurrence of the most damaging LULCTs, or mitigate their ecological impacts. The transition from pasture to cropland deserves particular attention, given the potential risks of intensifying the use of agrochemicals to freshwater ecosystems, which we did not document in this study. Fourth, we show that biodiversity is the ecosystem component most affected by land-use and land-cover change. While much of the world’s interest in tropical forest landscapes is around carbon emissions, we show that biodiversity requires increased attention, and hope this will be emphasized in the upcoming COP15 on Biodiversity and other global meetings. Finally, we show that understanding human influences in tropical forests requires going beyond single-component and binary assessments of the condition of the biome—by doing so, we hope that our assessment of a fuller range of transitions opens up new opportunities for landscape management.

## Materials and Methods

### Rates of LULCTs.

To estimate the rates of LULCTs in the Brazilian Amazon, we used data from two sources. First, for transitions from primary and secondary forests to pasture or agriculture, and from agriculture and pasture to secondary forest, we used land-use change maps based on the MapBiomas collection 5 dataset (1985 to 2019; mapbiomas.org) (15). We applied the method outlined in Smith et al. (30) to group the MapBiomas schema into four classes (forest, pasture, cropland, and other), and used a change-detection algorithm to further divide the forest class into primary and secondary forests (30). We further classified the secondary forests into two classes: “young” if they were ≤20 y old, and “old” if they were >20 y old. As the MapBiomas time series begins in 1985, any secondary forests that began growing before this date are included in the primary forest class. Using the threshold of 20 y, we just started to detect old secondary forests after 2005. Second, for transitions from undisturbed primary forests to logged or logged-and-burned primary forests, and from these primary forest types to deforested land-use types, we used forest degradation maps for the Brazilian Amazon from 1992 to 2014 (29), in which a digital object analysis framework and a digital spectral analysis were calibrated with field data to map forest disturbance by logging, fire, edge effects, and isolation (29). Based on these two datasets, we obtained information on 17 LULCTs (*SI Appendix*, Fig. S2) and calculated their mean annual rate (km^2^⋅y^−1^) for the periods 2006 to 2014 (forest degradation maps) and 2006 to 2019 (land-use change maps). We focus on LULCTs from 2006 onward because this year was a breaking point in the sociopolitical context in terms of land-use change in the Amazon (56), but the full time series of changes in the transition rates are presented in *SI Appendix*, Figs. S7 and S8. As the first dataset did not discriminate between undisturbed and degraded primary forests and the second dataset did not discriminate for which land use the primary forests were deforested, we estimated these annual rates. To do that, we used the ratio of the conversion from all primary forest to pasture and agriculture (0.985 to pasture and 0.015 to agriculture) to calculate the annual rate of the transitions from undisturbed, logged, and logged-and-burned primary forests to pasture and agriculture, based on their overall deforestation rates (*SI Appendix*, Fig. S2*B*). For instance, if we calculated a 6,070 km^2^⋅y^−1^ rate of undisturbed primary forest deforestation based on degradation maps (29), we estimated that 5,978.95 km^2^⋅y^−1^ were converted to pasture and 91.05 km^2^⋅y^−1^ were converted to agriculture. Thus, we evaluated 18 LULCT rates that were used to link the prevalence of transitions to their impact (*Linking Impacts with Transition Rates*).

### Impacts of LULCTs on Biodiversity, Carbon, and Soil.

#### Study sites and sampling design.

We collected data from 310 sites located in two regions of the eastern Amazon, in the Brazilian state of Pará: the municipalities of Santarém, Belterra, and Mojuí-dos-Campos (STM hereafter) and the municipality of Paragominas (PGM hereafter). All our study sites were located in places originally covered by terra firme forests. Both regions have experienced an increase in deforestation in the early 2000s, linked to the expansion of mechanized agriculture. The current landscape in these regions comprises a mosaic of undisturbed and disturbed primary forests, agricultural land uses, and secondary forests (48).

We separated both regions into third- or fourth-order drainage catchments (c. 5,000 ha each) and then selected 18 catchments in each region representing a gradient of forest cover. In each catchment, we installed 8 to 12 sites (transects or plots) separated by a minimum of 1.5 km to avoid spatial autocorrelation. Fauna sampling occurred along 300-m transects, while flora and soil properties were surveyed within 250 × 10–m plots (0.25 ha; *SI Appendix*, Fig. S1*A*). For more details on the sites and sampling design, see Gardner et al. (48).

Our 310 sites were distributed across seven different land-use and land-cover types (*SI Appendix*, Table S1): undisturbed primary forests; logged primary forests; logged-and-burned primary forests; old secondary forests (>20 y); young secondary forests (1 to 20 y old); pastures; and mechanized agriculture. The different types of land use and the ages of secondary forests were classified using field assessments combined with a time-series analysis of a chronosequence of satellite images (between 1988 and 2010 to 2011). For full details, see Gardner et al. (48).

#### Ecological variables.

We sampled 18 ecological variables to represent three different ecosystem components: biodiversity, carbon, and soil. We assessed biodiversity based on surveys of seven groups during 2010 and 2011. The diversity of plants was divided into three groups based on stem size or functional growth type: 1) large trees, 2) small trees, and 3) lianas, which were identified during the vegetation survey in the 0.25-ha plots (*SI Appendix*, Fig. S1*B*). To capture the diversity of fauna, 4) birds and 5) dung beetles were sampled at three points separated by 150 m along each transect (*SI Appendix*, Fig. S1*B*), 6) ants were sampled at six points separated by 50 m, and 7) orchid bees were sampled at five points, also separated by 50 m. More details on the sampling techniques for each taxon can be found in *SI Appendix*, *Methods* and in Gardner et al. (48). All variables were sampled in both regions, except for orchid bees, which were only sampled in PGM. We calculated total species richness and the richness of forest species (those species that occurred at least once at a primary forest site) for each biodiversity group at each sampling site.

We followed Intergovernmental Panel on Climate Change criteria (57) to separate ecosystem carbon stocks into the 1) above-ground carbon pool, 2) dead wood pool, 3) litter carbon pool, and 4) soil carbon pool. To obtain carbon stocks, we estimated biomass following Berenguer et al. (17) and full details of the calculations are given in *SI Appendix*, *Methods*. During the vegetation survey (in 2010 and 2011), we measured all individuals of live trees, palms, and lianas, as well as standing dead trees and palms with a diameter at breast height ≥10 cm. We first calculated the biomass of each sampled individual (*SI Appendix*, *Methods*) and then summed the values of all live trees, palms, and lianas to obtain the total above-ground biomass of each sampling site. The above-ground carbon pool was assumed to represent 50% of the total live biomass of each site (57). We also calculated the biomass of coarse woody debris (≥10 cm in diameter in at least one extremity) from measurements taken in five 0.01-ha subplots (5 × 20 m) in each study site. We then summed the biomass of standing dead trees and palms along with the biomass of coarse wood debris to obtain the dead wood carbon pool as 50% of the total dead biomass of each site [see Berenguer et al. (17) for the full methods and equations used]. We estimated the biomass of fine woody debris (2 to 10 cm in diameter in at least one extremity) from weighting all pieces found in a further five subplots measuring 2 × 5 m each (*SI Appendix*, Fig. S1*B*), while litter biomass was estimated from samples collected in ten 0.25-m^2^ quadrats per plot, which was later dried to a constant weight. The litter carbon pool was then calculated as the carbon content of fine woody debris and leaf litter. The soil carbon pool was the carbon stock measured at 0- to 30-cm depth (details below). All values were converted to carbon stocks per hectare.

To determine soil carbon stocks and soil properties, we collected soil samples in 10-cm increments from 0 to 30 cm at five sampling points, separated by 50 m, at each sampling site (*SI Appendix*, Fig. S1*B*) and bulked them to give one composite sample per site and depth. The soil sampling took place during the carbon stock surveys in 2010 and 2011. We measured bulk density using two intact soil cores per depth increment, which were collected in the center of each plot using a volumetric ring. Soil carbon content and chemical properties were subsequently analyzed using dried soils following standard methods (58) for pH, nitrogen, phosphorus, potassium, calcium + magnesium, sodium, and aluminum and following Ellertl and Bettany (59) for soil organic carbon. Soil carbon stocks were then calculated by multiplying the carbon content of each layer by the layer thickness (10 cm) and the bulk density of the soil, and were adjusted to compare equivalent masses of soil between different land uses. Further details on soil sampling and analyses are given in Gardner et al. (48) and in Durigan et al. (60).

Finally, to account for confounding effects of soil type and topography when analyzing the LULCTs, we obtained the clay content from each soil sample using the densimeter method (61) and the mean elevation and slope in a 100-m buffer around each site using digital elevation models (48).

#### Data analysis.

All data analyses were conducted in R version 4.1.0 (62), and the R codes and data (63) are available at Zenodo (https://doi.org/10.5281/zenodo.6347560), and at GitHub (https://github.com/cassioalencarnunes/LULC_transitions) (64). Based on our 18 ecological variables in seven land-use and land-cover types, we evaluated 18 LULCTs, of which three were bidirectional (i.e., pastures to mechanized agriculture, and both pastures and mechanized agriculture to young secondary forests). To do that, we first centered (subtracted the variable mean of each value) and scaled (divided each value by the SD of the variable) all our response and explanatory variables using the 'scale' function. Then, we ran linear mixed models (LMMs) using the 'lmer' function from the lme4 package (65), with each ecological variable as the response variable, the land-use and land-cover classes (categorical variable with seven levels) as the explanatory variable, and clay content, elevation, and slope as covariates. In addition, we also included in all models the catchment ID and region (STM and PGM) as random factors, except for the model of orchid bee species richness, which only included catchment ID, as this taxon was sampled only in the PGM region. To evaluate which LULCT had an effect on each response variable, we performed multiple comparisons of means of land-use and land-cover classes (15 comparisons, for each unidirectional transition and one for each of the three bidirectional transitions) using the function 'glht' from the multcomp package (66). To limit type I error, the contrasts were made using single-step tests, which accounts for correlation between the test statistics and adjusts the *P* value considering the multiple comparisons. In addition, we also calculated the simultaneous CI for each contrast (i.e., each transition) considering the multiple comparisons using the 'confint' function. We considered the transition as having a significant influence on a response variable at *P* < 0.05 and used the beta coefficient to obtain the direction of the relationship. All models were checked for overdispersion and homoscedasticity using the functions 'simulatedResiduals', 'testDispersion', and 'testCategorical' from the DHARMa package (67) and by checking both the Q-Q and the boxplots of residuals for each land-use and land-cover class (*SI Appendix*, Figs. S9–S11). The χ^2^ and *P* values for each model and the results of model diagnostics are shown in *SI Appendix*, Table S5.

For a limited subset of the models, the Q-Q plots revealed small deviations from the normal distribution and unequal variances among the land-use and land-cover classes. This occurred in models where a preponderance of zeros in some of the response categories (e.g., tree species richness in pastures and mechanized agriculture) inevitably resulted in very low variance within these land-use classes. Although the deviations from normal distribution were minor, we validated all models using quantile generalized additive models (qGAMs) to test the sensitivity of our results to the deviations from normal distribution and unequal variances (68). We followed the same steps of LMM analysis and performed multiple comparisons of medians (0.5 quantile) to compute effect sizes and *P* values. We then ran correlation tests to compare the effect sizes obtained from the qGAMs with the effect sizes obtained using LMMs. This validation demonstrated the insensitivity of our findings to deviations from the normal distribution and unequal variances. First, the results of all significant models were highly similar (*r* = 0.99), validating our LMMs. Second, of the 270 pairwise comparisons (18 variables × 15 transitions), 258 (95.5%) remained the same side of the significance threshold (0.05). Of the transitions that changed their significance (*SI Appendix*, Table S7), most (3.7%, *n* = 10) revealed transitions that became significant using the qGAM, and only 0.75% (*n* = 2) became nonsignificant with the qGAM. There was no clear pattern in these changes in significance, and they occurred in both models that met all assumptions of good fit and in models that presented deviations from normal distribution and unequal variances. The full description and results of the validation analysis are given in *SI Appendix*, *Methods* and Fig. S12.

### Transition Magnitude of Change on the Ecosystem.

#### Effect size analysis.

To understand the magnitude of changes on biodiversity, carbon, and soil, we used standardized effect sizes. The standardized effect sizes and their CIs were the coefficient and 95% CI generated by the multiple comparisons of means described above. As we centered and scaled all variables, the difference of mean estimated values of land-use and land-cover classes in the LMMs can be interpreted as the effect size of each transition (each comparison) on each variable. These effect sizes are also comparable among different variables that originally had different units (e.g., between ecosystem components). The effect sizes (and CI) of each variable and for each LULCT are presented in *SI Appendix*, Table S6.

To compare the LULCTs, we used the absolute values of the effect sizes (i.e., transformed negative into positive values), because here we were interested in the magnitude of change but not in its direction. We plotted the absolute values of the effect sizes of all variables by each transition separated by ecosystem components. Then, we ranked the transitions by their median effect sizes—the median of the effect on seven, four, and seven variables of biodiversity, carbon, and soil, respectively. We considered the median effect size of each transition as representing its “average” effect on the ecosystem component.

### Linking Impacts with Transition Rates.

To better understand the ecological impacts of LULCTs, we analyzed the relationship between the annual rates of each LULCT and the median effect size of each ecosystem component (biodiversity, carbon, and soil) by running correlation tests ('cor.test' function). For biodiversity and soil ecosystem components we ran the Pearson’s correlation test, and for the carbon component we ran the Spearman’s rank correlation test, as the carbon median effect sizes did not follow a normal distribution. In addition, we divided our data into four classes representing a scale of increasing impact and rate for each LULCT: 1) low impact, low rate, 2) low impact, high rate, 3) high impact, low rate, and 4) high impact, high rate. To achieve this, we calculated the median annual LULCT rates and median effect sizes for each ecosystem component and used the median values as cutoff points to distinguish low and high rates or impacts.

## Supplementary Material

Supplementary File

## Data Availability

Data and code reported in this article have been deposited in Zenodo (https://doi.org/10.5281/zenodo.6347560) (63), and GitHub (https://github.com/cassioalencarnunes/LULC_transitions) (64). All other study data are included in the article and/or *SI Appendix*.
