# Supplementary material for: Linking land-use and land-cover transitions to their ecological impact in the Amazon
Source: Proc Natl Acad Sci U S A. 2022 Jun 27;119(27):e2202310119. doi: 10.1073/pnas.2202310119 (PMC9271202; doi:10.1073/pnas.2202310119)
Supplement: Supplementary File [file pnas.2202310119.sapp.pdf]

PNAS
www.pnas.org

Supplementary Information for "Linking land-use and land-cover transitions to their ecological impact in the Amazon"

Cássio Alencar Nunes, Erika Berenguer, Filipe França, Joice Ferreira, Alexander C. Lees, Julio Louzada, Emma J. Sayer, Ricardo Solar, Charlotte Smith, Luiz E. O. C. Aragão, Danielle de Lima Braga, Plinio Camargo, Carlos Cerri, Raimundo Cosme, Mariana Durigan, Nárgila Moura, Victor Hugo Fonseca Oliveira, Carla Ribas, Fernando Vaz-de-Mello, Ima Vieira, Ronald Zanetti, Jos Barlow

Cássio Alencar Nunes
Email: cassioalencarnunes@gmail.com

This PDF file includes:

Supplementary Methods
Figures S1 to S12
Tables S1 to S7
SI References

Supplementary Methods

Detailed information on above-ground biomass and biodiversity sampling

Information on biomass estimation

To estimate tree biomass, species-specific wood density was obtained from the Global Wood Density Database (1). If a species-specific wood density was not available, the average of the genus or family was used. For all trees and saplings, we used the allometric equation of Chave et al (2) to calculate biomass based on diameter at breast height (DBH) and wood density, except for the genus *Cecropia* for which we used the Nelson allometric equation (3). We used the Gerwing & Farias equation (4) to estimate the biomass of lianas.

Biomass estimation of litter components (fine wood debris and leaf litter) was made by weighing the samplings after drying to constant weight. For coarse woody debris, each piece of dead wood was classified in terms of its decomposition state, and biomass was calculated by multiplying its volume by its density. More details on biomass estimation can be found in refs (5, 6).

Detailed information of animal sampling

Birds: Sampling was conducted at three points along each transect separated by 150 m (0, 150 and 300 m). At each of these three points, 15-minute point count samples were collected between 15 minutes before dawn and 9:30 am. These surveys were repeated at each point in the transect, but in reverse order to remove temporal bias. Solid-state sound recorders were used to record each point count survey to facilitate identification of any species not identified in the field. See ref (7) and ref (8) for details on the bird survey and a list of voucher species collected in Santarém and Paragominas, respectively.

Ants: We used passive (non-baited) pitfall traps in the Santarém region, whereas in Paragominas, the traps were baited with sardine and honey. Traps were plastic containers (12 cm height and 8 cm diameter) half-filled with a solution of water, salt (2%) and detergent (5%), and were left in the field for 48 h. In Paragominas, six traps were installed per transect, separated by 50 m (between 25 and 275 m in each transect). In Santarém, 10 traps were installed along each transect separated by 10 m (between 50 – 90 and 200 – 240 m in each transect). All collected specimens were kept in ethanol and then were taken to the Universidade Federal de Lavras and Universidade Federal de Viçosa – Minas Gerais – Brazil, where they were identified to the lowest taxonomic level. Final identification was checked by an expert taxonomist (Dr. Rodrigo Feitosa at the Museu de Zoologia de São Paulo). For a full list of voucher species collected in Paragominas see ref (9).

Dung Beetles: We used baited pitfall traps, which were 1-litre plastic containers (4 cm diameter and 9 cm height) with a bait container filled with 50 g of dung (80% pig and 20% human). All traps were half-filled with a solution of water, salt (2%) and detergent (5%). Dung beetles were sampled at three points along each transect separated by 150 m (0, 150 and 300 m). In each of these three sampling points, 3 pitfalls were installed in the corners of a triangle (3-m per side) and were left in the field for 48 h. The individuals were kept in ethanol for preservation and taken to the Universidade Federal de Lavras and Universidade Federal de Viçosa – Minas Gerais – Brazil for identification. Final identification was checked by an expert taxonomist (Dr. Fernando Zagury Vaz-de-Mello at the Universidade Federal do Mato Grosso).

Orchid bees: we collected male orchid bees using baited traps, made of 2-litre plastic bottles (0 cm diameter and 35 cm height). Traps were installed at four sampling points along each transect in the Paragominas region, and were separated by 50 m (50, 100, 150 and 200 m). In each bottle, radial holes were made at 20 cm height, where we inserted flower-like structures that were lined with coarse sand to give support to the bees. Inside the bottle, a stick with cotton ball was baited with eugenol, methyl salicylate, vanilla or eucalyptol (each trap received only one type of bait). These traps were tied to tree trunks 1.5 m above the ground and were left in the field for 48 h. Bees were kept frozen before sorting out and identification by an expert taxonomist (Dr. André Nemésio at EMBRAPA – Amazônia Oriental). For a full list of voucher species collected see ref (9).

Model validation analysis

Visual inspection of diagnostic plots revealed that some of our linear mixed-effect models (LMMs) presented residuals with deviations from a normal distribution, outlier influence or unequal variances (Fig. S9, S10 and S11). Most of these violations were relatively small and a result of low variance in certain response variables for pastures and mechanized agriculture land-use and land-cover (LULC) classes. Nonetheless, we ran a validation analysis to verify that the results of our LMMs were robust.

First, we ran non-parametric Quantile Generalized Additive Models (qGAM) using the function '*qgam*' from R package 'qgam' (10, 11). We used the standardized ecosystem variables as response variables and the LULC classes as the explanatory variable, together with mean elevation, mean slope and clay content as co-variates (i.e., the same model structure as our LMMs). We also included catchment ID and region as smoothed random factors in the qGAMs. The main difference between the qGAMs and LMMs was that we constructed the qGAM models based on the 0.5 quantile (i.e., the median), whereas the LMMs compared means. Second, we ran the simultaneous comparisons of median values in the different LULC class, using contrast matrices and accounting for inflation of type I error (in the same way as for LMMs). We used the functions '*glht*' and '*confint*' from 'multcomp' package (12) to obtain the standardized effect sizes and their confidence intervals for each LULC transition.

Finally, to validate our LMMs, we compared the effect sizes obtained using qGAMs with the effect sizes obtained using the LMM approach. We ran correlation tests using the base-R function 'cor.test'. The tests revealed that the effect sizes were highly correlated, with most of the *r* coefficients = 0.99 (Fig. S12). The only two models with lower *r* coefficients were for the soil carbon pool and soil Na, with $r = 0.88$ and $r = 0.90$, respectively. Crucially, these were non-significant models, so the weaker correlation does not affect the results. In terms of significance (p-value), of the 270 pairwise comparisons (18 variables x 15 transitions), 258 (95.5%) remained the same side of the significance threshold (0.05). Of the transitions that changed their significance (Table S7), most (3.7%, n = 10) revealed transitions that became significant using the qGAM, and only 0.75% (n = 2) became non-significant with the qGAM. There was no clear pattern in these changes in significance, and they occurred in both models that met all assumptions of goof fit and in models that presented deviations from normal distribution and unequal variances. Thus, we confirmed that the LMMs with residuals deviating slightly from a normal distribution and unequal variances between LULC were indeed robust, strengthening our results and conclusions.

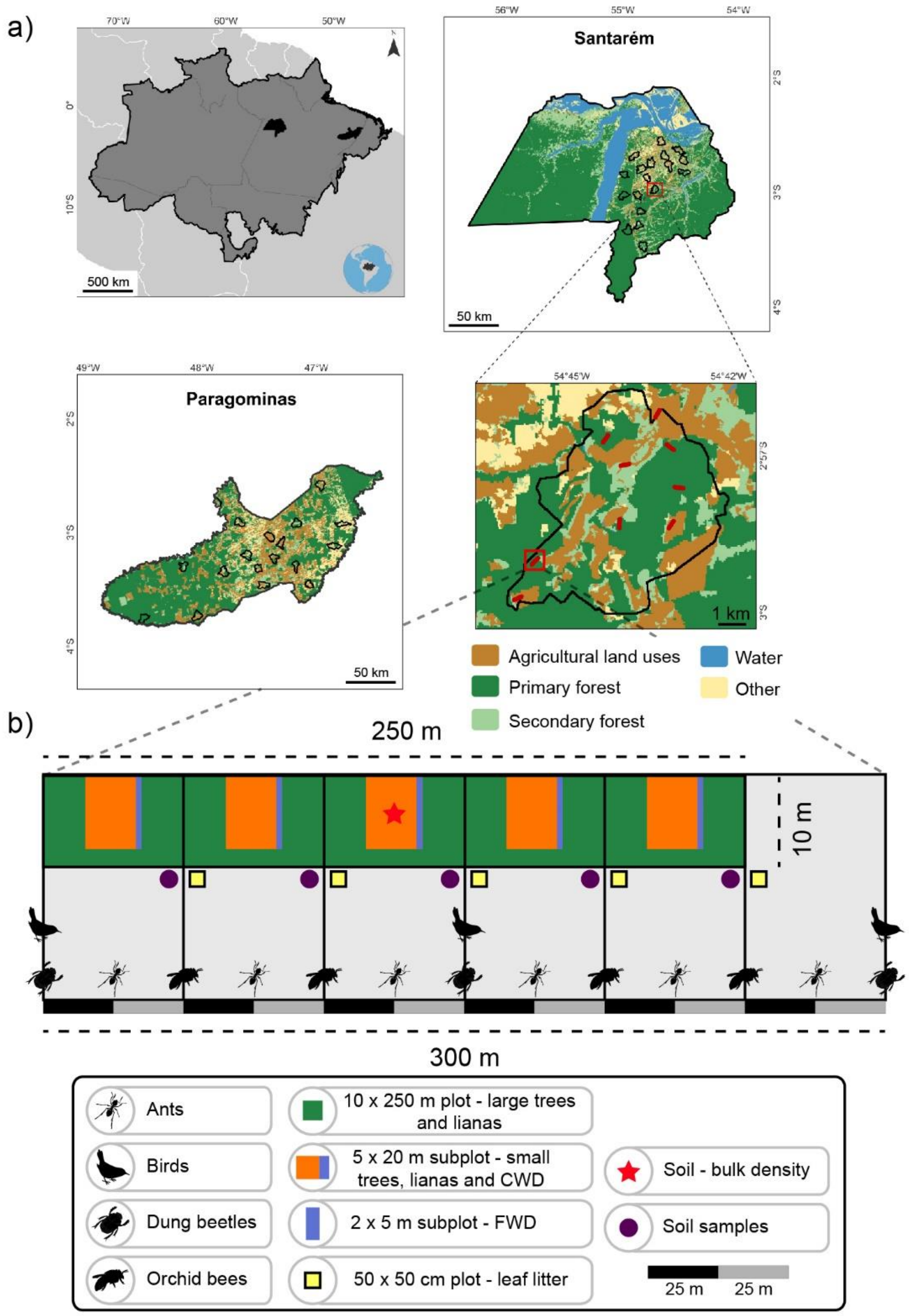

Fig. S1. Study sites and design. **(a)** Map of the study sites in the Santarém (STM) and Paragominas (PGM) regions of the eastern Amazon, in the Brazilian state of Pará. **(b)** Diagram of plots, subplots, and sampling design points to collect ecological variables representing biodiversity, carbon pools and soil properties at the study sites, where CWD = coarse woody debris and FWD = fine woody debris.

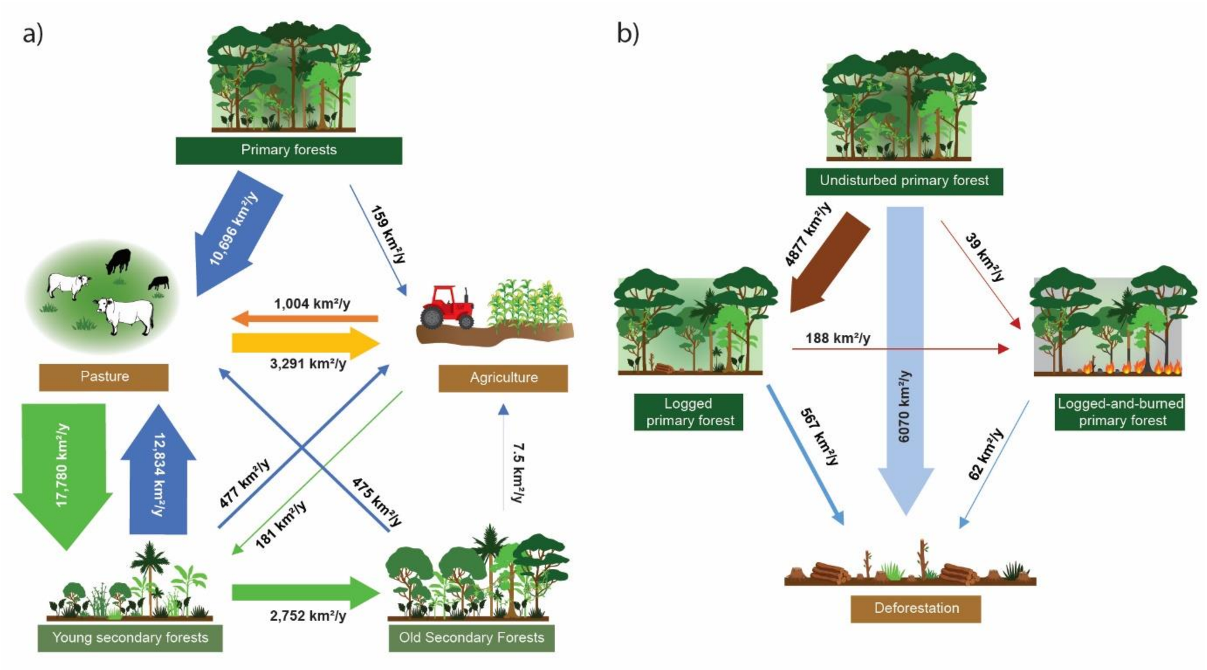

Fig. S2. Land use and cover transition (LULCT) rates in the Brazilian Amazon. Mean annual LULCT rates from (a) 2006-2019, based on land-use change maps (13) and (b) 2006-2014, based on forest degradation maps (14). Primary forests have never been clear cut, and secondary forests are regenerating forests. Young secondary forests are < 20 years old and old secondary forests are ≥ 20 years old. Agriculture includes perennial and temporary crops. The width of the arrows is proportional to the mean annual rate of the transition.

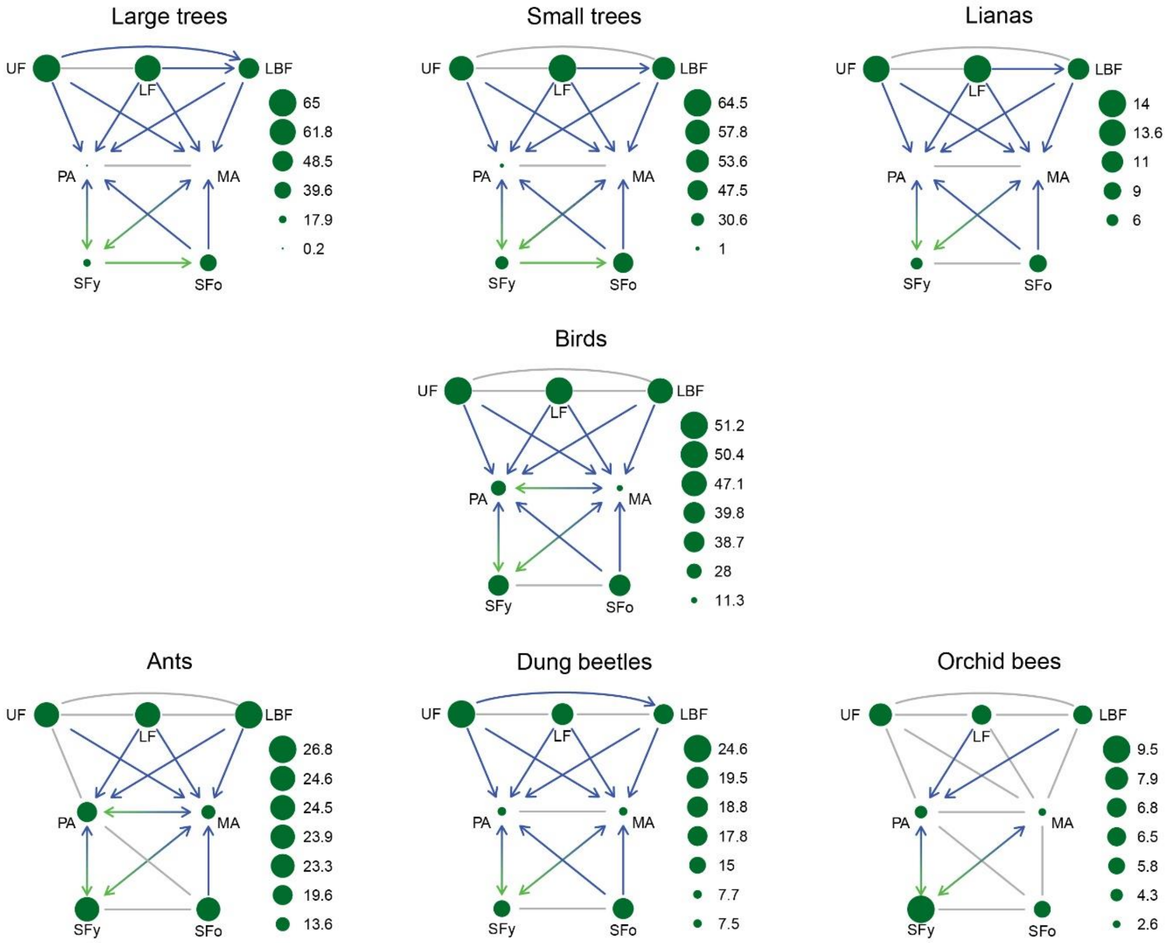

Fig. S3. Impacts of 18 land-use and land-cover transitions on the species richness of seven biodiversity groups in the Brazilian Amazon. Values and size of symbols represent the species richness averages based on 21 undisturbed primary forests (UF), 68 logged primary forests (LF), 65 logged-and-burned primary forests (LBF), 72 pastures (PA), 26 mechanized agriculture fields (MA), 33 young secondary forests (SFy, < 20 years old) and 25 old secondary forests (SFo, ≥ 20 years old). Arrows indicate the transitions and their effect, where grey indicates no effect, green is a significant increase and blue a significant decrease.

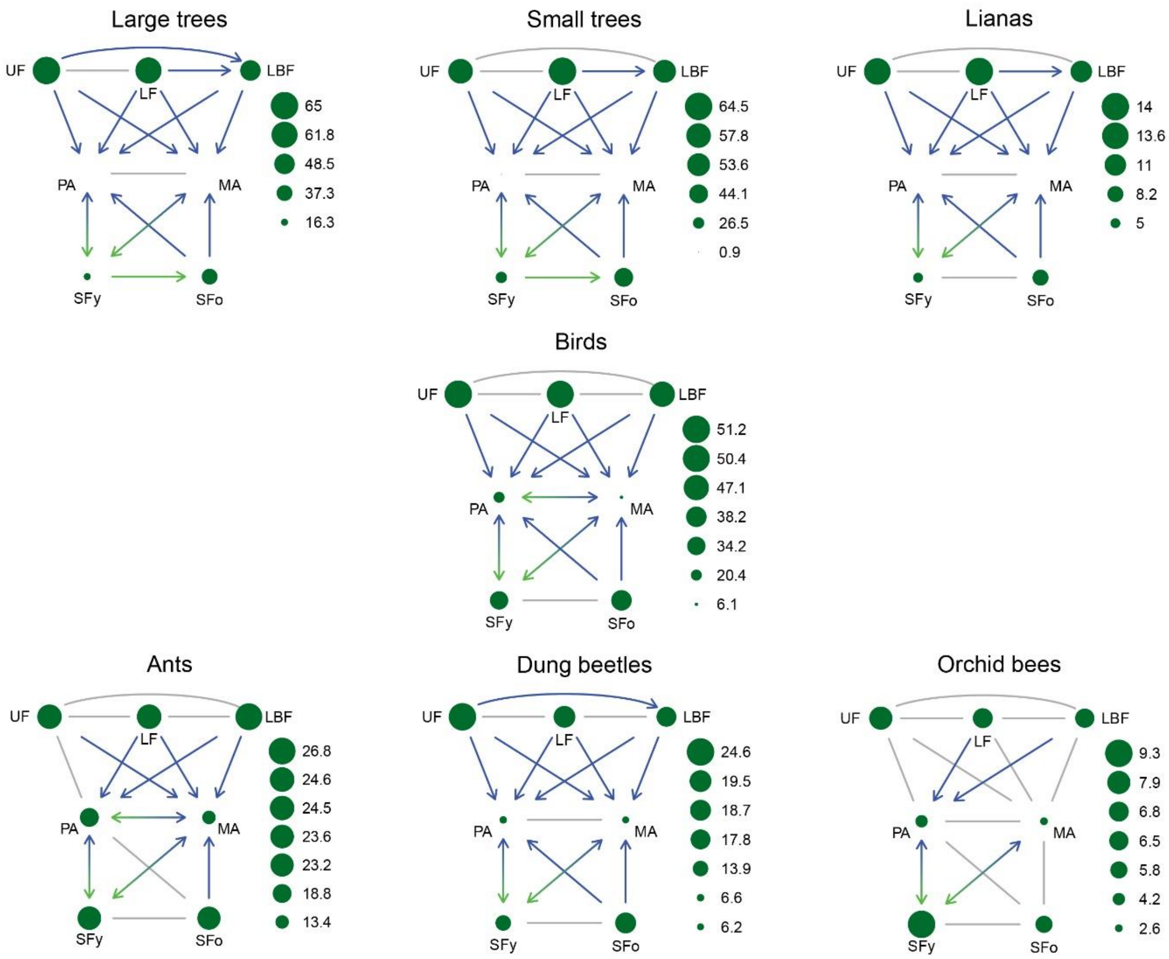

Fig. S4. Impacts of 18 land-use and land-cover transitions on the forest species richness of seven biodiversity groups in the Brazilian Amazon. Values and size of symbols represent the species richness averages based on 21 undisturbed primary forests (UF), 68 logged primary forests (LF), 65 logged-and-burned primary forests (LBF), 72 pastures (PA), 26 mechanized agriculture fields (MA), 33 young secondary forests (SFy, < 20 years old) and 25 old secondary forests (SFo, ≥ 20 years old). Arrows indicate the transitions and their effect, where grey indicates no effect, green is a significant increase and blue a significant decrease.

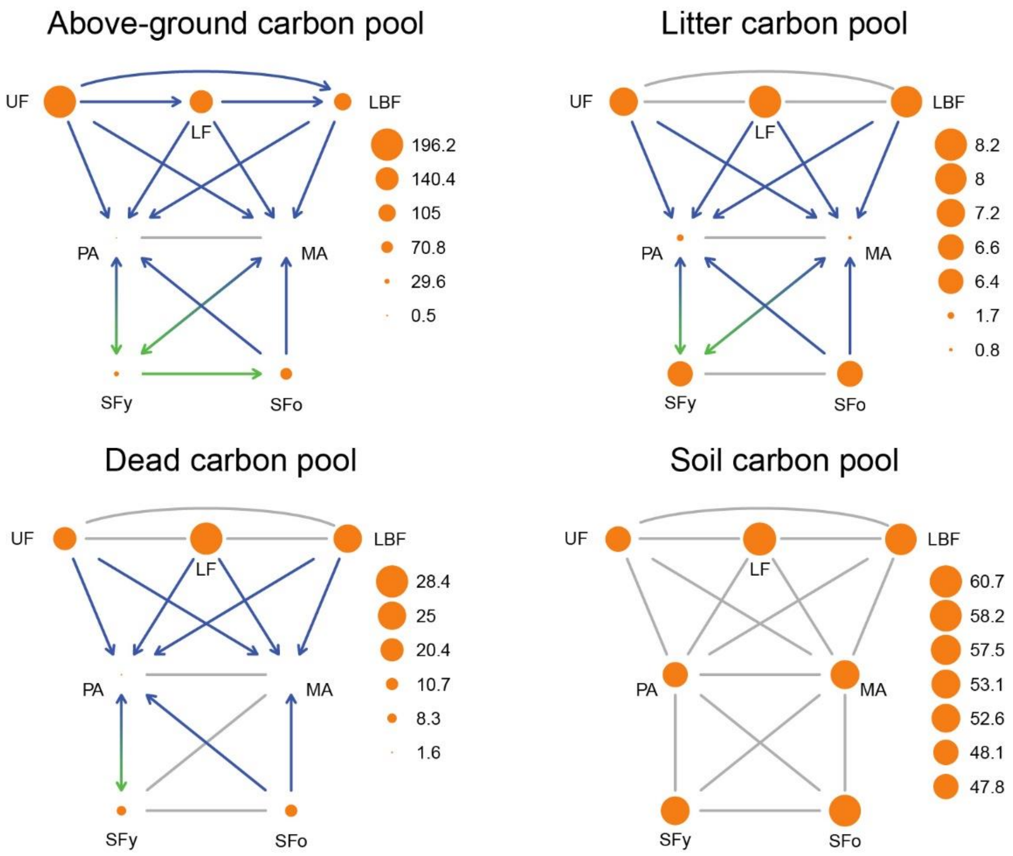

Fig. S5. Impacts of 18 land-use and land-cover transitions on four ecosystem carbon pools in the Brazilian Amazon. Values and size of symbols represent the averages of carbon pools in Mg ha^{-1} based on 21 undisturbed primary forests (UF), 68 logged primary forests (LF), 65 logged and burned primary forests (LBF), 72 pastures (PA), 26 mechanized agriculture fields (MA), 33 young secondary forests (SFy, < 20 years old) and 25 old secondary forests (SFo, ≥ 20 years old). Arrows indicate the transitions and their effect, where grey indicates no effect, green is a significant increase and blue a significant decrease.

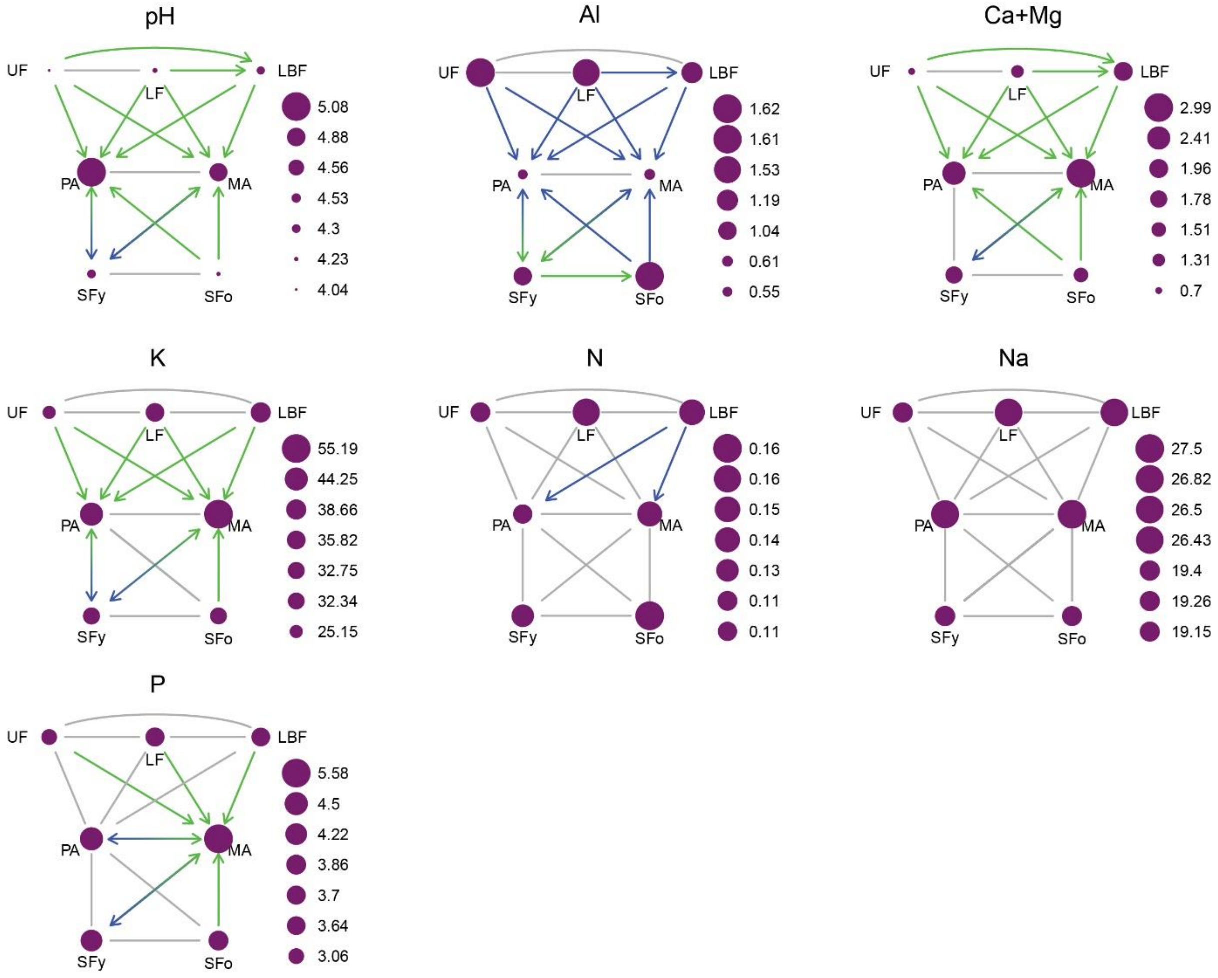

Fig. S6. Impacts of 18 land-use and land-cover transitions on seven soil properties at 0-10 cm depth in the Brazilian Amazon. Values represent the averages based on 21 undisturbed primary forests (UF), 68 logged primary forests (LF), 65 logged-and-burned primary forests (LBF), 72 pastures (PA), 26 mechanized agriculture fields (MA), 33 young secondary forests (SFy, < 20 years old) and 25 old secondary forests (SFo, ≥ 20 years old). Arrows indicate the transitions and their effect, where grey indicates no effect, green is a significant increase and blue a significant decrease. The size of symbols indicates nutrient concentrations or pH, where Al: Aluminium (mmolc dm^{-3}), Ca+Mg: Calcium + Magnesium (mmolc dm^{-3}), K: Potassium (mg dm^{-3}), N: Nitrogen (%), Na: Sodium (mg dm^{-3}), P: Phosphorus (mg dm^{-3}).

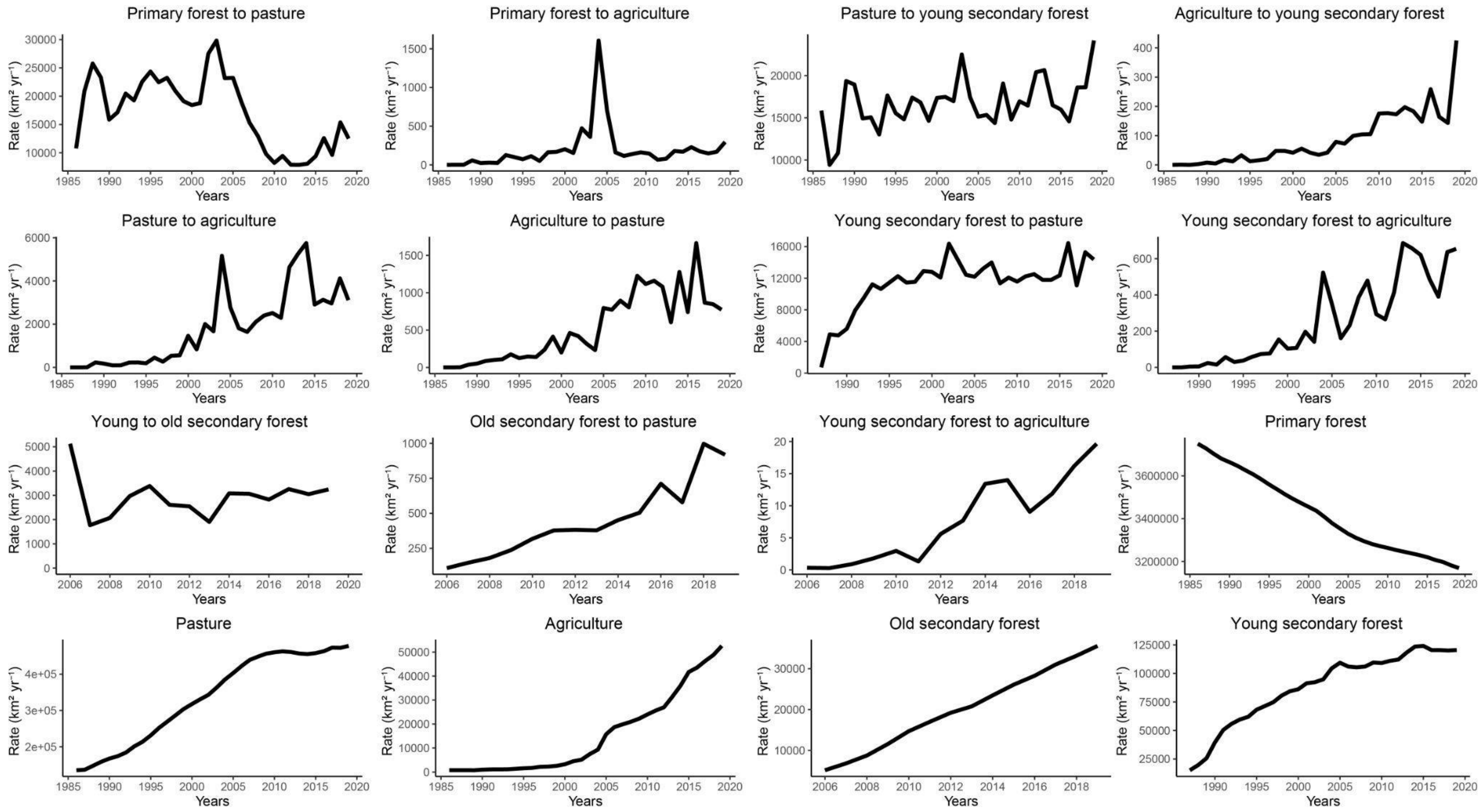

Fig. S7. Annual rates (km² yr^{-1}) of land-use and land-cover transitions. Data for the Brazilian Amazon from 1985 to 2019, based on the MapBiomas collection 5 dataset (13).

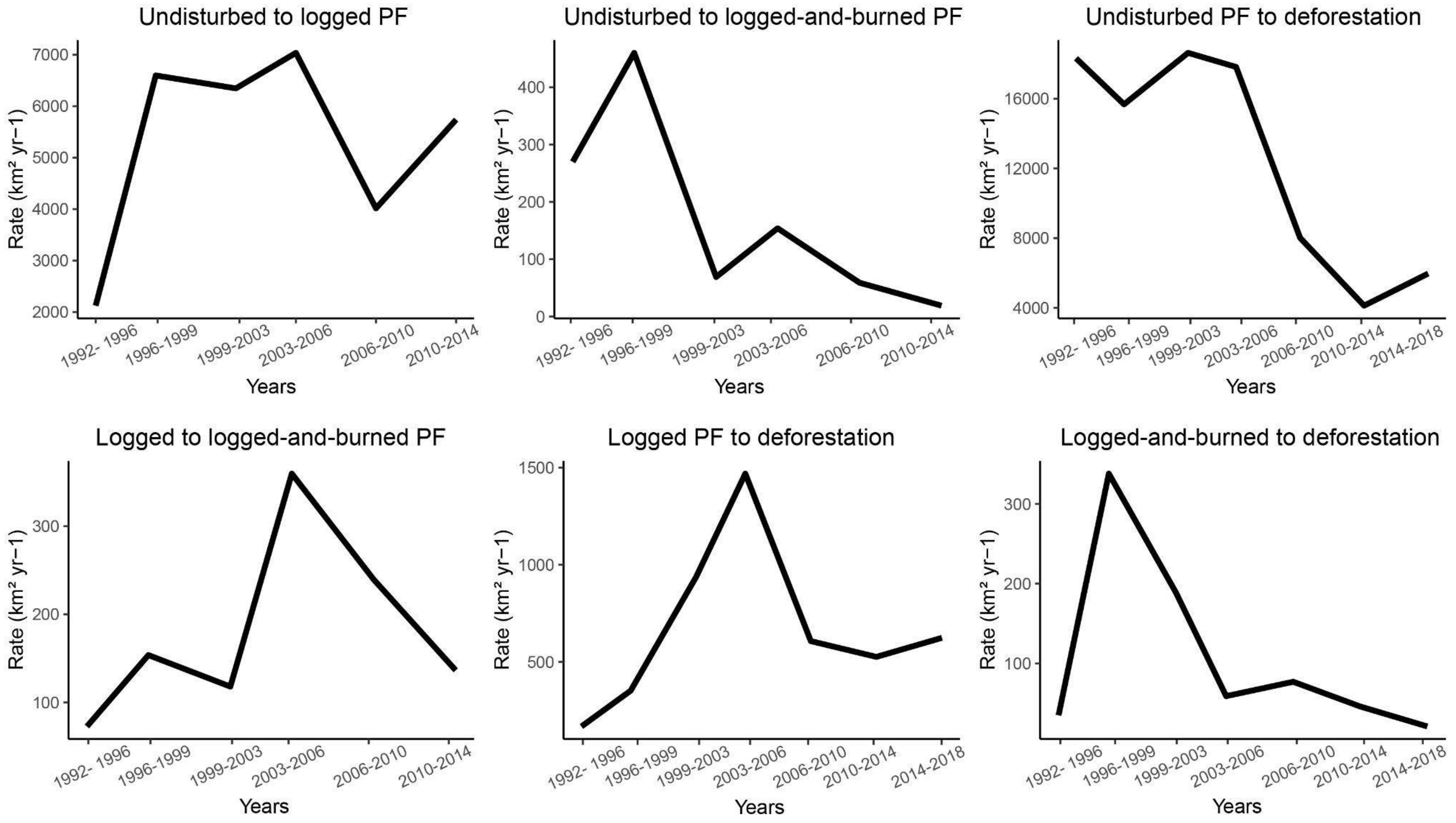

Fig. S8. Annual rates (km² yr^{-1}) of land-use and land-cover transitions related to primary forest degradation and deforestation. Data for the Brazilian Amazon from 1992 to 2018, based on (14). The rates are the mean values for the intervals shown on the X axis.

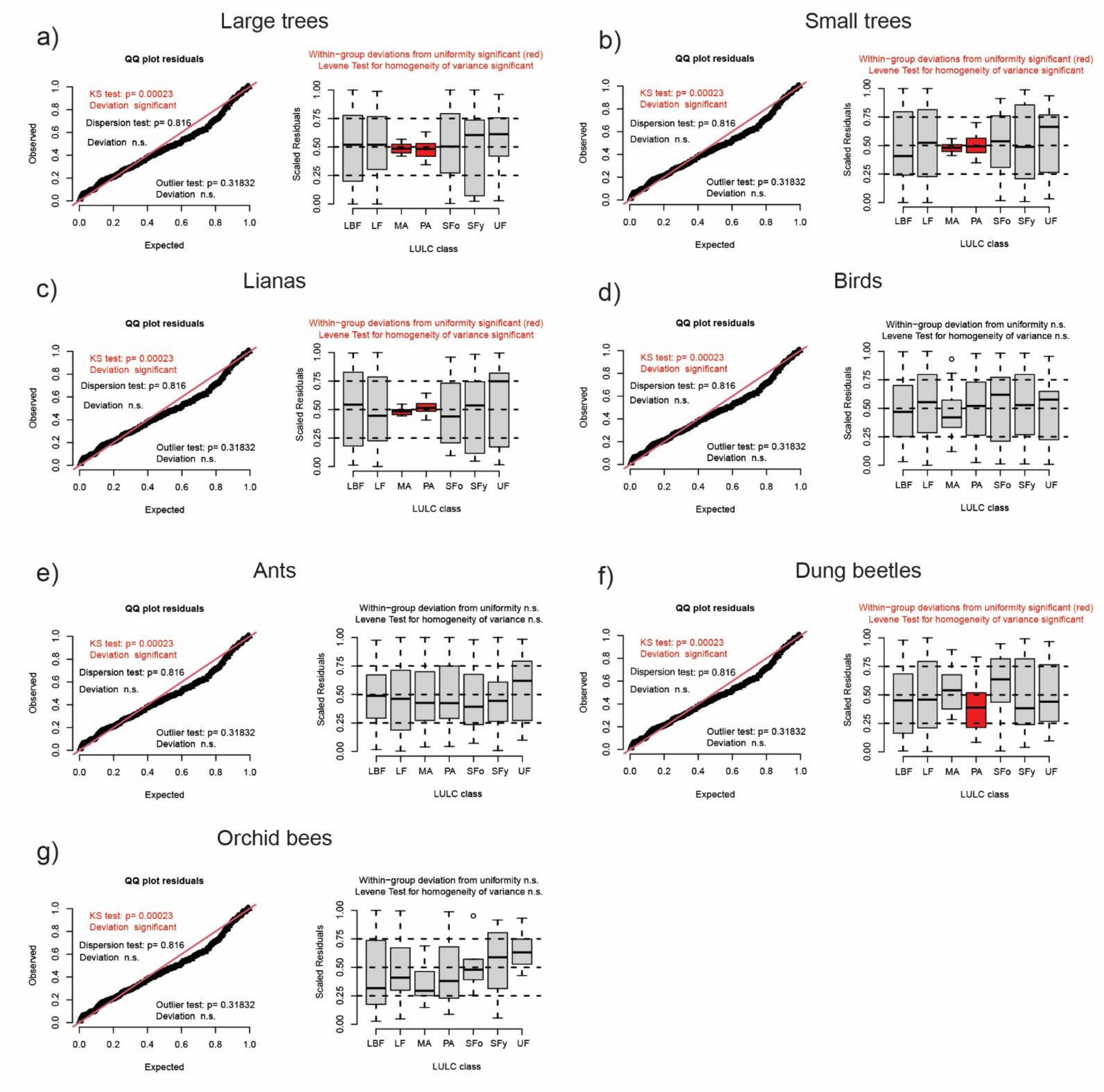

Fig. S9. Model diagnostics for variables of the biodiversity ecosystem component. We checked all models for both overdispersion and homoscedasticity using the package DHARMa in R (10, 15). When variance was not homogeneous, we checked the boxplots of residuals per land-use and land-cover class (LULC) to decide about the adequacy of the models. Biodiversity models included species richness as response variables. The number of replicates are 21 undisturbed primary forests (UF), 68 logged primary forests (LF), 65 logged-and-burned primary forests (LBF), 72 pastures (PA), 26 mechanized agriculture fields (MA), 33 young secondary forests (SFy, < 20 years old) and 25 old secondary forests (SFo, ≥ 20 years old).

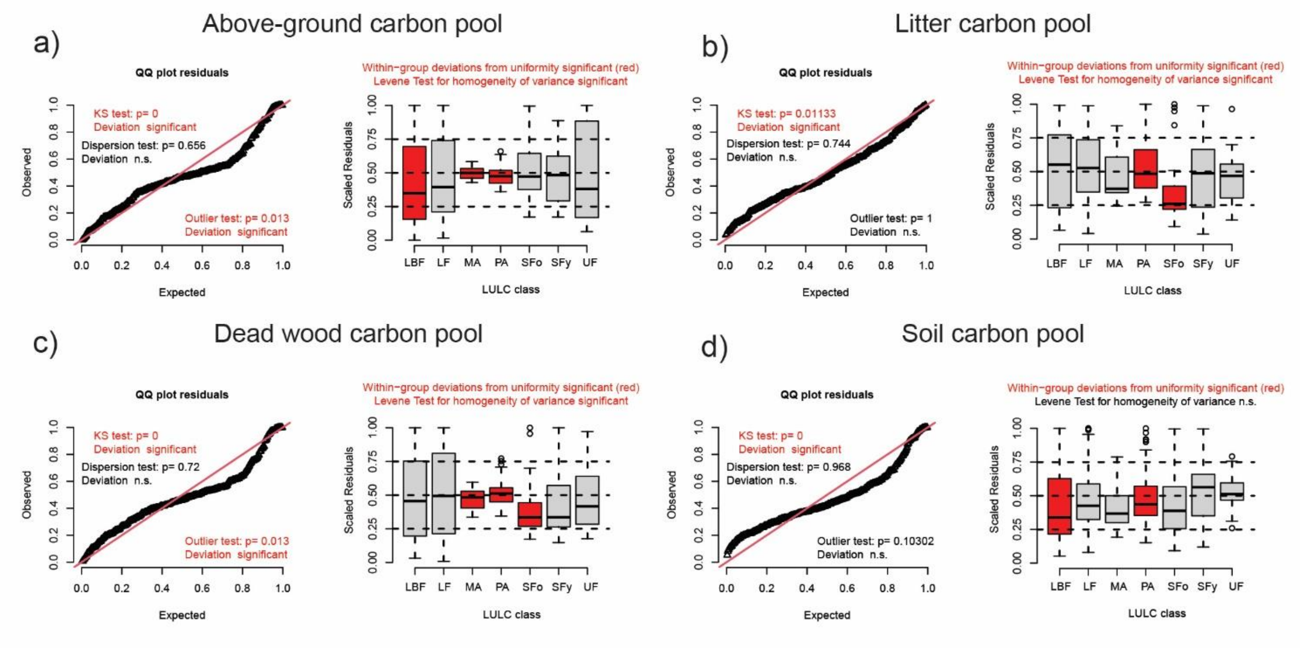

Fig. S10. Model diagnostics for variables of the carbon ecosystem component. We checked all models for both overdispersion and homoscedasticity using the package DHARMa in R (10, 15). When variance was not homogeneous, we checked the boxplots of residuals per land-use and land-cover class (LULC) to decide about the adequacy of the models. Carbon models included the values of carbon pools in Mg ha^{-1} as response variables. The number of replicates are 21 undisturbed primary forests (UF), 68 logged primary forests (LF), 65 logged-and-burned primary forests (LBF), 72 pastures (PA), 26 mechanized agriculture fields (MA), 33 young secondary forests (SFy, < 20 years old) and 25 old secondary forests (SFo, ≥ 20 years old).

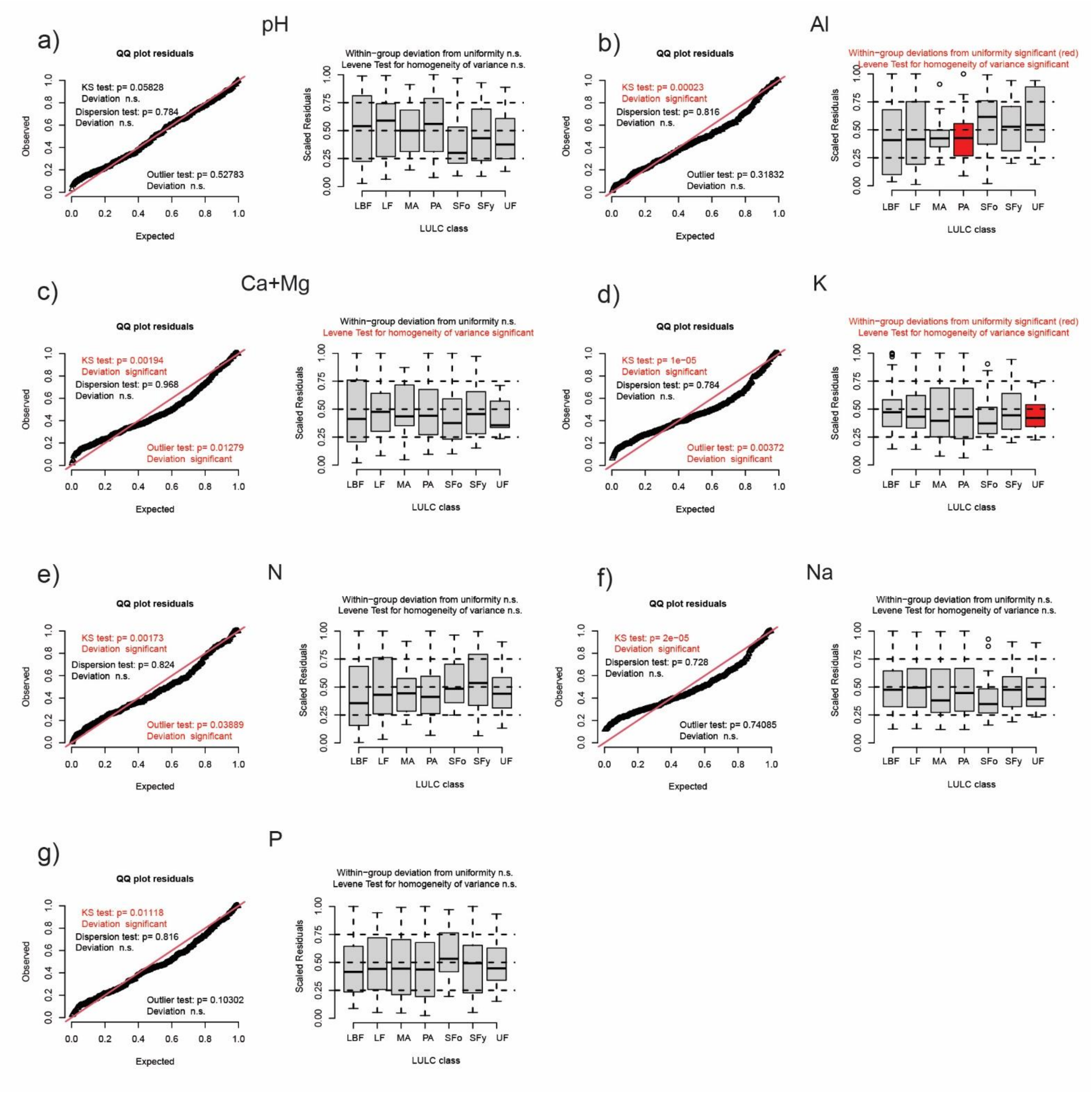

Fig. S11. Model diagnostics for variables of the soil ecosystem component. We checked all models for both overdispersion and homoscedasticity using the package DHARMa in R (10, 15). When variance was not homogeneous, we checked the boxplots of residuals per land-use and land-cover class (LULC) to decide about the adequacy of the models. Soil models included the values of nutrient concentrations as response variables; Al: Aluminium (mmolc dm^{-3}), Ca+Mg: Calcium + Magnesium (mmolc dm^{-3}), K: Potassium (mg dm^{-3}), N: Nitrogen (%), Na: Sodium (mg dm^{-3}), P: Phosphorus (mg dm^{-3}). The number of replicates are 21 undisturbed primary forests (UF), 68 logged primary forests (LF), 65 logged-and-burned primary forests (LBF), 72 pastures (PA), 26 mechanized agriculture fields (MA), 33 young secondary forests (SFy, < 20 years old) and 25 old secondary forests (SFo, ≥ 20 years old).

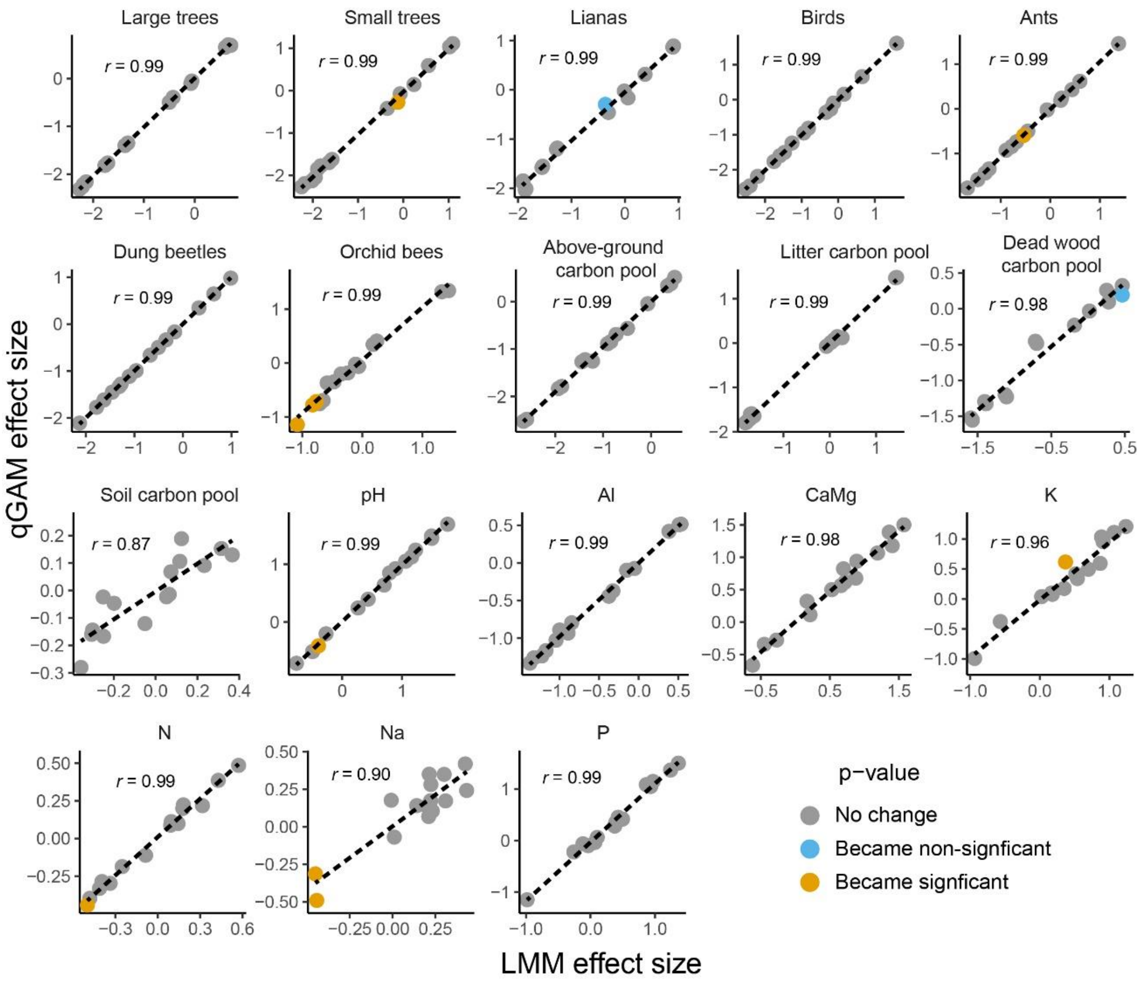

Fig. S12. Results of model validation analysis showing the relationship between the standardized effect sizes for each LULC transition calculated using linear mixed-effect models (LMM) on the X axis and the standardized effect sizes obtained using quantile generalized additive models (qGAM) on the Y axis. Circle colors show the changes in significance when comparing the two approaches (LMM with qGAM). The correlation coefficients (*r*) are shown for each response variable.

Table S1. Number of sampling sites of each land-use and land-cover type in the two sampling regions in the Brazilian Amazon, State of Pará.

Region	Santarém (STM)	Paragominas (PGM)
Undisturbed primary forest	12	9
Logged primary forest	25	43
Logged-and-burned primary forest	23	42
Old secondary forest	20	5
Young secondary forest	18	15
Pasture	23	49
Mechanized agriculture	16	10
**Total**	**137**	**173**

Table S2. Mean annual rates ($km^2\ yr^{-1}$) of land-use and land-cover transitions (LULCT) in the Brazilian Amazon and their standard deviation (Sd). The rates were calculated and estimated (*) based on land-use change maps (2006-2019; (13)) and on forest degradation maps (2006-2014; (14)).

LULCT	Rate ($km^2\ yr^{-1}$)	Sd Rate
UF - LF	4877	1218
UF - LBF	39	28
UF - PA*	5981	2711
UF - MA*	88	40
LF - LBF	188	72
LF - PA*	559	56
LF - MA*	8	0.8
LBF - PA*	61	21
LBF - MA*	1	0.3
PA - MA	3291	1263
MA - PA	1004	286
PA - SFy	17780	2840
SFy - PA	12834	1657
MA - SFy	181	85
SFy - MA	477	161
SFo - PA	475	264
SFo - MA	7.5	6.6
SFy - SFo	2752	536

Table S3. Mean values of the 18 ecological variables sampled in 310 transects over two regions in the Brazilian Amazon, State of Pará. The values are shown for each land-use or land-cover type. Units for biodiversity variables is species richness, for carbon pools is Mg ha^{-1}, and for soil variables are Al and Ca+Mg = mmolc dm^{-3}, K, Na and P = mg dm^{-3}, and N is %. UF: Undisturbed primary forest, LF: Logged primary forest, LBF: Logged-and-burned primary forest, SFo: Old secondary forest, SFy: Young secondary forest, PA: Pasture, MA: Mechanized agriculture.

Ecosystem component	Variable	UF	LF	LBF	PA	MA	SFy	SFo
**Biodiversity**	Bird	51.24	50.35	47.12	27.96	11.35	38.67	39.84
	Ant	24.48	24.57	26.83	19.56	13.62	23.90	23.32
	Dung beetle	24.57	19.49	17.75	7.65	7.50	15.03	18.76
	Orchid bee	7.88	6.76	6.52	4.29	2.60	9.47	5.80
	Liana	13.57	14.03	11.02	0.04	0.00	6.03	8.96
	Small tree	57.81	64.51	53.63	0.96	0.00	30.61	47.48
	Large trees	65.00	61.79	48.49	0.19	0.00	17.88	39.60
**Carbon**	Above-ground pool	196.15	140.36	105.00	0.48	0.00	29.64	70.83
	Litter pool	7.24	8.23	8.01	1.69	0.84	6.44	6.60
	Dead wood pool	20.44	28.45	25.02	1.62	0.00	8.26	10.73
	Soil pool	48.13	60.71	57.48	47.83	52.55	53.07	58.17
**Soil**	pH	4.04	4.30	4.53	5.08	4.88	4.56	4.23
	N	0.11	0.16	0.15	0.11	0.14	0.13	0.16
	P	3.06	3.70	3.64	4.50	5.58	4.22	3.86
	K	25.15	35.82	38.66	44.25	55.19	32.75	32.34
	Na	19.26	26.50	26.43	26.82	27.50	19.40	19.15
	CaMg	0.70	1.31	1.96	2.41	2.99	1.78	1.51
	Al	1.62	1.53	1.19	0.55	0.61	1.04	1.61

Table S4. Percentage changes in the mean values of 18 ecological variables in response to 18 land-use and land-cover transitions (LULCT) in the Brazilian Amazon. Positive values represent an increase in the variable, while negative values represent the percentage of decrease. An increase above 100% is represented as a multiplier and marked in bold. NA values are increases from 0, making it impossible to give percentages. Biodiversity is the species richness of the listed groups. UF: Undisturbed primary forest, LF: Logged primary forest, LBF: Logged-and-burned primary forest, SFo: Old secondary forest, SFy: Young secondary forest, PA: Pasture, MA: Mechanized agriculture.

	**Biodiversity**							**Carbon stocks**				**Soil properties**						
**LULCT**	**Bird**	**Ant**	**Dung beetle**	**Orchid bee**	**Liana**	**Small tree**	**Large tree**	**Above-ground pool**	**Litter pool**	**Dead wood pool**	**Soil pool**	**pH**	**N**	**P**	**K**	**Na**	**CaMg**	**Al**
UF - PA	-45.4	-20.1	-68.9	-45.5	-99.7	-98.3	-99.7	-99.8	-76.7	-92.1	-0.6	25.6	-2.8	47.2	75.9	39.3	**3.4x**	-66.1
LF - PA	-44.5	-20.4	-60.7	-36.5	-99.7	-98.5	-99.7	-99.7	-79.5	-94.3	-21.2	18.0	-29.3	21.6	23.5	1.2	83.8	-63.9
LBF - PA	-40.7	-27.1	-56.9	-34.2	-99.6	-98.2	-99.6	-99.5	-78.9	-93.5	-16.8	12.1	-24.9	23.8	14.5	1.5	22.8	-53.9
UF - MA	-77.9	-44.4	-69.5	-67.0	-100.0	-100.0	-100.0	-100.0	-88.3	-100.0	9.2	20.6	26.2	82.6	**2.2x**	42.8	**4.2x**	-62.3
LF - MA	-77.5	-44.6	-61.5	-61.5	-100.0	-100.0	-100.0	-100.0	-89.7	-100.0	-13.4	13.4	-8.2	50.9	54.0	3.8	**2.2x**	-59.9
LBF - MA	-75.9	-49.3	-57.8	-60.1	-100.0	-100.0	-100.0	-100.0	-89.5	-100.0	-8.6	7.7	-2.6	53.5	42.7	4.1	52.4	-48.8
SFo - PA	-29.8	-16.1	-59.2	-26.0	-99.5	-98.0	-99.5	-99.3	-74.4	-84.9	-17.8	20.0	-32.9	16.7	36.8	40.0	59.4	-65.7
SFo - MA	-71.5	-41.6	-60.0	-55.2	-100.0	-100.0	-100.0	-100.0	-87.2	-100.0	-9.7	15.2	-13.0	44.7	70.6	43.6	97.8	-61.9
SFy - PA	-27.7	-18.2	-49.1	-54.7	-99.3	-96.9	-98.9	-98.4	-73.8	-80.4	-9.9	11.5	-14.6	6.6	35.1	38.2	35.4	-47.1
SFy - MA	-70.7	-43.0	-50.1	-72.5	-100.0	-100.0	-100.0	-100.0	-86.9	-100.0	-1.0	7.0	10.8	32.3	68.5	41.7	68.0	-41.2
SFy - SFo	3.0	-2.4	24.8	-38.7	48.6	55.1	**2.2x**	**2.4x**	2.5	30.0	9.6	-7.1	27.3	-8.6	-1.2	-1.3	-15.1	54.2
PA - SFy	38.3	22.2	96.4	**2.2x**	**144x**	**31.9x**	**91.9x**	**61.6x**	**3.8x**	**4.1x**	11.0	-10.3	17.1	-6.2	-26.0	-27.7	-26.1	89.0
MA - SFy	**3.6x**	75.6	**2x**	**3.6**	NA	NA	NA	NA	663.2	NA	1.0	-6.6	-9.8	-24.4	-40.7	-29.4	-40.5	70.1
PA - MA	-59.4	-30.4	-2.0	-39.4	-100.0	-100.0	-100.0	-100.0	-50.0	-100.0	9.9	-4.0	29.8	24.0	24.7	2.5	24.1	11.1
MA - PA	**2.5x**	43.6	2.0	65.1	NA	NA	NA	NA	99.8	NA	-9.0	4.1	-22.9	-19.4	-19.8	-2.5	-19.4	-10.0
UF - LF	-1.7	0.4	-20.7	-14.1	3.4	11.6	-4.9	-28.4	13.6	39.2	26.1	6.4	37.5	21.1	42.4	37.6	87.3	-6.1
UF - LBF	-8.0	9.6	-27.7	-17.2	-18.8	-7.2	-25.4	-46.5	10.6	22.4	19.4	12.1	29.5	19.0	53.7	37.3	**2.8x**	-26.4
LF - LBF	-6.4	9.2	-8.9	-3.5	-21.5	-16.9	-21.5	-25.2	-2.7	-12.0	-5.3	5.3	-5.8	-1.7	7.9	-0.3	49.7	-21.7

Table S5. Complete list of model χ², degrees of freedom (Df), p-values and p-values for overdispersion and homoscedasticity tests. Small trees = DAP < 10 cm, large trees = DAP ≥ 10 cm. LULC: land-use and land-cover, CLAY: clay content, ELEV_MEAN: mean elevation of transect (m.a.s.l), SLOPE_MEAN: mean slope of transect.

Response variable	Explanatory variable	χ²	Df	p-value	Overdispersion p-value	Homoscedasticity p-value
**Ant richness**	LULC	100.3368	6	2.13E-19	0.752	0.753138
	CLAY	0.172815	1	0.677622		
	ELEV_MEAN	0.004249	1	0.94803		
	SLOPE_MEAN	0.256351	1	0.612638		
**Bird richness**	LULC	406.5227	6	1.11E-84	0.672	0.065008
	CLAY	0.028974	1	0.864839		
	ELEV_MEAN	4.527974	1	0.033345		
	SLOPE_MEAN	1.436592	1	0.230692		
**Dung beetle richness**	LULC	284.9986	6	1.34E-58	0.912	0.000186
	CLAY	0.011486	1	0.914653		
	ELEV_MEAN	0.518982	1	0.471276		
	SLOPE_MEAN	0.616124	1	0.432491		
**Liana richness**	LULC	444.3362	6	8.13E-93	0.648	4.47E-33
	CLAY	1.699701	1	0.192327		
	ELEV_MEAN	0.097227	1	0.755183		
	SLOPE_MEAN	0.481449	1	0.487767		
**Small tree richness**	LULC	1364.927	6	9.51E-292	0.688	3.75E-25
	CLAY	3.536004	1	0.06005		
	ELEV_MEAN	0.420983	1	0.516447		
	SLOPE_MEAN	0.712497	1	0.398616		
**Large tree richness**	LULC	1617.519	6	0	0.704	4.66E-26
	CLAY	3.329426	1	0.068051		
	ELEV_MEAN	0.462132	1	0.496629		
	SLOPE_MEAN	1.557342	1	0.212055		
**Orchid bee richness**	LULC	43.72737	6	8.37E-08	0.288	0.185288
	CLAY	2.513656	1	0.112864		
	ELEV_MEAN	2.444479	1	0.117939		
	SLOPE_MEAN	2.903707	1	0.088376		
**Above-ground pool**	LULC	833.6231	6	8.36E-177	0.656	1.74E-22
	CLAY	1.77274	1	0.183043		
	ELEV_MEAN	0.273139	1	0.601234		
	SLOPE_MEAN	3.343365	1	0.067476		
**Litter pool**	LULC	693.7814	6	1.35E-146	0.744	3.90E-05
	CLAY	1.768255	1	0.183598		
	ELEV_MEAN	0.8651	1	0.352316		
	SLOPE_MEAN	0.065645	1	0.797786		
**Dead wood pool**	LULC	223.1351	6	2.23E-45	0.72	9.48E-19

	CLAY	3.245399	1	0.071624		
	ELEV_MEAN	5.216955	1	0.022368		
	SLOPE_MEAN	0.20385	1	0.651631		
**Soil pool**	LULC	7.28475	6	0.295315	0.968	0.056227
	CLAY	34.03451	1	5.41E-09		
	ELEV_MEAN	2.151768	1	0.142406		
	SLOPE_MEAN	9.541173	1	0.002009		
**pH**	LULC	238.7315	6	1.05E-48	0.784	0.098647
	CLAY	0.705146	1	0.40106		
	ELEV_MEAN	11.05097	1	0.000886		
	SLOPE_MEAN	1.586378	1	0.207844		
**N**	LULC	24.93997	6	0.00035	0.824	0.100848
	CLAY	88.1105	1	6.19E-21		
	ELEV_MEAN	8.776362	1	0.003052		
	SLOPE_MEAN	2.284987	1	0.130631		
**P**	LULC	34.83536	6	4.64E-06	0.816	0.220176
	CLAY	3.597972	1	0.05785		
	ELEV_MEAN	5.961557	1	0.014621		
	SLOPE_MEAN	1.322648	1	0.250117		
**K**	LULC	52.94086	6	1.21E-09	0.784	0.002305
	CLAY	11.3088	1	0.000771		
	ELEV_MEAN	5.486538	1	0.019163		
	SLOPE_MEAN	0.274401	1	0.600395		
**Na**	LULC	11.09669	6	0.085434	0.728	0.242018
	CLAY	8.101901	1	0.004422		
	ELEV_MEAN	4.191095	1	0.040637		
	SLOPE_MEAN	0.664525	1	0.414967		
**CaMg**	LULC	105.577	6	1.72E-20	0.968	0.00704
	CLAY	4.656576	1	0.030935		
	ELEV_MEAN	13.24981	1	0.000273		
	SLOPE_MEAN	3.49965	1	0.061382		
**Al**	LULC	170.8554	6	2.96E-34	0.816	2.97E-08
	CLAY	28.72315	1	8.35E-08		
	ELEV_MEAN	9.916299	1	0.001638		
	SLOPE_MEAN	3.628776	1	0.056789		

Table S6. Effect sizes, the lower and upper confidence interval (CI), the p-value and the standard error (Std error) of the effect size of all land-use and land-cover transitions (LULCT) on the different ecological variables of biodiversity, carbon, and soil ecosystem components. The column “Effect” shows whether the LULCT had a positive, negative or no effect on a given response variable. Small trees = DAP < 10 cm, large trees = DAP ≥ 10 cm. UF: Undisturbed primary forest, LF: Logged primary forest, LBF: Logged-and-burned primary forest, SFo: Old secondary forest, SFy: Young secondary forest, PA: Pasture, MA: Mechanized agriculture.

LULCT	Variable	Effect Size	CI lower	CI upper	p-value	Std error	Effect	Component
UF - LF	**Ant richness**	0.229	-0.501	0.959	0.964	0.257	no effect	biodiversity
UF - LBF		0.440	-0.338	1.217	0.615	0.274	no effect	biodiversity
UF - PA		-0.453	-1.228	0.323	0.579	0.273	no effect	biodiversity
UF - MA		-1.234	-2.102	-0.365	0.001	0.306	negative	biodiversity
LF - LBF		0.211	-0.218	0.640	0.755	0.151	no effect	biodiversity
LF - PA		-0.682	-1.117	-0.246	0.000	0.153	negative	biodiversity
LF - MA		-1.463	-2.030	-0.895	0.000	0.200	negative	biodiversity
LBF - PA		-0.892	-1.315	-0.469	0.000	0.149	negative	biodiversity
LBF - MA		-1.673	-2.223	-1.124	0.000	0.194	negative	biodiversity
PA - MA		-0.781	-1.343	-0.219	0.001	0.198	negative	biodiversity
PA - SFy		0.599	0.097	1.101	0.009	0.177	positive	biodiversity
MA - SFy		1.380	0.737	2.023	0.000	0.226	positive	biodiversity
SFo - PA		-0.535	-1.091	0.021	0.068	0.196	no effect	biodiversity
SFo - MA		-1.316	-1.970	-0.662	0.000	0.230	negative	biodiversity
SFy - SFo		-0.064	-0.679	0.551	1.000	0.217	no effect	biodiversity
UF - LF	**Bird richness**	-0.125	-0.627	0.378	0.989	0.177	no effect	biodiversity
UF - LBF		-0.333	-0.851	0.185	0.462	0.182	no effect	biodiversity
UF - PA		-1.597	-2.115	-1.080	0.000	0.182	negative	biodiversity
UF - MA		-2.540	-3.136	-1.944	0.000	0.209	negative	biodiversity
LF - LBF		-0.208	-0.533	0.117	0.468	0.114	no effect	biodiversity
LF - PA		-1.472	-1.802	-1.143	0.000	0.116	negative	biodiversity
LF - MA		-2.416	-2.845	-1.986	0.000	0.151	negative	biodiversity
LBF - PA		-1.264	-1.588	-0.941	0.000	0.114	negative	biodiversity
LBF - MA		-2.207	-2.637	-1.778	0.000	0.151	negative	biodiversity
PA - MA		-0.943	-1.378	-0.509	0.000	0.153	negative	biodiversity
PA - SFy		0.656	0.273	1.039	0.000	0.134	positive	biodiversity
MA - SFy		1.599	1.109	2.090	0.000	0.172	positive	biodiversity
SFo - PA		-0.816	-1.250	-0.382	0.000	0.153	negative	biodiversity
SFo - MA		-1.759	-2.267	-1.251	0.000	0.179	negative	biodiversity
SFy - SFo		0.160	-0.318	0.638	0.951	0.168	no effect	biodiversity
UF - LF	**Dung beetle richness**	-0.505	-1.044	0.035	0.083	0.190	no effect	biodiversity
UF - LBF		-0.674	-1.254	-0.095	0.012	0.204	negative	biodiversity
UF - PA		-1.781	-2.357	-1.204	0.000	0.203	negative	biodiversity
UF - MA		-2.131	-2.771	-1.491	0.000	0.225	negative	biodiversity
LF - LBF		-0.170	-0.473	0.134	0.627	0.107	no effect	biodiversity
LF - PA		-1.276	-1.584	-0.968	0.000	0.109	negative	biodiversity
LF - MA		-1.626	-2.028	-1.225	0.000	0.141	negative	biodiversity
LBF - PA		-1.106	-1.405	-0.808	0.000	0.105	negative	biodiversity

LBF - MA		-1.457	-1.842	-1.071	0.000	0.136	negative	biodiversity
PA - MA		-0.350	-0.745	0.045	0.119	0.139	no effect	biodiversity
PA - SFy		0.635	0.289	0.981	0.000	0.122	positive	biodiversity
MA - SFy		0.985	0.536	1.434	0.000	0.158	positive	biodiversity
SFo - PA		-0.963	-1.355	-0.572	0.000	0.138	negative	biodiversity
SFo - MA		-1.313	-1.773	-0.854	0.000	0.162	negative	biodiversity
SFy - SFo		0.328	-0.098	0.754	0.246	0.150	no effect	biodiversity
UF - LF	**Liana richness**	0.055	-0.425	0.535	1.000	0.169	no effect	biodiversity
UF - LBF		-0.312	-0.805	0.182	0.485	0.173	no effect	biodiversity
UF - PA		-1.850	-2.343	-1.356	0.000	0.173	negative	biodiversity
UF - MA		-1.864	-2.434	-1.295	0.000	0.200	negative	biodiversity
LF - LBF		-0.367	-0.679	-0.054	0.011	0.110	negative	biodiversity
LF - PA		-1.905	-2.221	-1.589	0.000	0.111	negative	biodiversity
LF - MA		-1.919	-2.332	-1.506	0.000	0.145	negative	biodiversity
LBF - PA		-1.538	-1.849	-1.227	0.000	0.109	negative	biodiversity
LBF - MA		-1.552	-1.966	-1.139	0.000	0.145	negative	biodiversity
PA - MA		-0.014	-0.432	0.404	1.000	0.147	no effect	biodiversity
PA - SFy		0.892	0.524	1.260	0.000	0.129	positive	biodiversity
MA - SFy		0.906	0.435	1.378	0.000	0.166	positive	biodiversity
SFo - PA		-1.266	-1.684	-0.849	0.000	0.147	negative	biodiversity
SFo - MA		-1.281	-1.770	-0.791	0.000	0.172	negative	biodiversity
SFy - SFo		0.374	-0.086	0.834	0.191	0.162	no effect	biodiversity
UF - LF	**Small tree richness**	0.233	-0.079	0.546	0.283	0.110	no effect	biodiversity
UF - LBF		-0.121	-0.441	0.199	0.913	0.112	no effect	biodiversity
UF - PA		-1.944	-2.264	-1.624	0.000	0.113	negative	biodiversity
UF - MA		-2.014	-2.387	-1.641	0.000	0.131	negative	biodiversity
LF - LBF		-0.354	-0.562	-0.147	0.000	0.073	negative	biodiversity
LF - PA		-2.177	-2.387	-1.967	0.000	0.074	negative	biodiversity
LF - MA		-2.247	-2.520	-1.974	0.000	0.096	negative	biodiversity
LBF - PA		-1.823	-2.029	-1.616	0.000	0.073	negative	biodiversity
LBF - MA		-1.893	-2.168	-1.618	0.000	0.097	negative	biodiversity
PA - MA		-0.070	-0.348	0.208	0.988	0.098	no effect	biodiversity
PA - SFy		1.019	0.775	1.264	0.000	0.086	positive	biodiversity
MA - SFy		1.089	0.776	1.403	0.000	0.110	positive	biodiversity
SFo - PA		-1.574	-1.850	-1.297	0.000	0.097	negative	biodiversity
SFo - MA		-1.644	-1.969	-1.318	0.000	0.114	negative	biodiversity
SFy - SFo		0.554	0.248	0.861	0.000	0.108	positive	biodiversity
UF - LF	**Large tree richness**	-0.075	-0.369	0.219	0.987	0.103	no effect	biodiversity
UF - LBF		-0.500	-0.805	-0.195	0.000	0.107	negative	biodiversity
UF - PA		-2.212	-2.518	-1.907	0.000	0.107	negative	biodiversity
UF - MA		-2.268	-2.617	-1.918	0.000	0.123	negative	biodiversity
LF - LBF		-0.425	-0.612	-0.238	0.000	0.066	negative	biodiversity
LF - PA		-2.137	-2.326	-1.948	0.000	0.067	negative	biodiversity
LF - MA		-2.192	-2.439	-1.945	0.000	0.087	negative	biodiversity

LBF - PA		-1.712	-1.898	-1.527	0.000	0.065	negative	biodiversity
LBF - MA		-1.767	-2.012	-1.522	0.000	0.086	negative	biodiversity
PA - MA		-0.055	-0.304	0.193	0.994	0.087	no effect	biodiversity
PA - SFy		0.607	0.388	0.825	0.000	0.077	positive	biodiversity
MA - SFy		0.662	0.381	0.943	0.000	0.099	positive	biodiversity
SFo - PA		-1.318	-1.566	-1.070	0.000	0.087	negative	biodiversity
SFo - MA		-1.373	-1.663	-1.083	0.000	0.102	negative	biodiversity
SFy - SFo		0.711	0.439	0.983	0.000	0.096	positive	biodiversity
UF - LF	**Orchid bee richness**	0.241	-1.054	1.537	0.998	0.460	no effect	biodiversity
UF - LBF		0.179	-1.180	1.538	1.000	0.482	no effect	biodiversity
UF - PA		-0.474	-1.811	0.863	0.934	0.474	no effect	biodiversity
UF - MA		-0.587	-2.135	0.962	0.911	0.549	no effect	biodiversity
LF - LBF		-0.062	-0.587	0.463	1.000	0.186	no effect	biodiversity
LF - PA		-0.715	-1.247	-0.184	0.002	0.188	negative	biodiversity
LF - MA		-0.828	-1.692	0.036	0.069	0.306	no effect	biodiversity
LBF - PA		-0.653	-1.175	-0.131	0.005	0.185	negative	biodiversity
LBF - MA		-0.766	-1.571	0.040	0.073	0.286	no effect	biodiversity
PA - MA		-0.113	-0.991	0.766	1.000	0.312	no effect	biodiversity
PA - SFy		1.324	0.645	2.004	0.000	0.241	positive	biodiversity
MA - SFy		1.437	0.424	2.451	0.001	0.359	positive	biodiversity
SFo - PA		-0.242	-1.308	0.825	0.993	0.378	no effect	biodiversity
SFo - MA		-0.354	-1.674	0.965	0.983	0.468	no effect	biodiversity
SFy - SFo		-1.083	-2.197	0.031	0.062	0.395	no effect	biodiversity
UF - LF	**Above-ground pool**	-0.731	-1.101	-0.360	0.000	0.130	negative	carbon
UF - LBF		-1.220	-1.597	-0.843	0.000	0.132	negative	carbon
UF - PA		-2.596	-2.972	-2.219	0.000	0.132	negative	carbon
UF - MA		-2.663	-3.102	-2.225	0.000	0.154	negative	carbon
LF - LBF		-0.489	-0.736	-0.243	0.000	0.087	negative	carbon
LF - PA		-1.865	-2.114	-1.616	0.000	0.087	negative	carbon
LF - MA		-1.933	-2.258	-1.608	0.000	0.114	negative	carbon
LBF - PA		-1.376	-1.621	-1.130	0.000	0.086	negative	carbon
LBF - MA		-1.443	-1.772	-1.114	0.000	0.116	negative	carbon
PA - MA		-0.068	-0.399	0.263	0.996	0.116	no effect	carbon
PA - SFy		0.346	0.054	0.638	0.010	0.103	positive	carbon
MA - SFy		0.414	0.041	0.787	0.019	0.131	positive	carbon
SFo - PA		-0.840	-1.172	-0.508	0.000	0.117	negative	carbon
SFo - MA		-0.908	-1.296	-0.519	0.000	0.136	negative	carbon
SFy - SFo		0.494	0.128	0.861	0.002	0.129	positive	carbon
UF - LF	**Litter pool**	0.163	-0.231	0.558	0.873	0.139	no effect	carbon
UF - LBF		0.092	-0.309	0.493	0.993	0.141	no effect	carbon
UF - PA		-1.658	-2.058	-1.257	0.000	0.141	negative	carbon
UF - MA		-1.635	-2.105	-1.165	0.000	0.165	negative	carbon
LF - LBF		-0.071	-0.333	0.190	0.982	0.092	no effect	carbon
LF - PA		-1.821	-2.085	-1.556	0.000	0.093	negative	carbon
LF - MA		-1.798	-2.148	-1.449	0.000	0.123	negative	carbon

LBF - PA		-1.750	-2.011	-1.489	0.000	0.092	negative	carbon
LBF - MA		-1.727	-2.081	-1.374	0.000	0.124	negative	carbon
PA - MA		0.022	-0.335	0.380	1.000	0.126	no effect	carbon
PA - SFy		1.445	1.135	1.756	0.000	0.109	positive	carbon
MA - SFy		1.423	1.022	1.824	0.000	0.141	positive	carbon
SFo - PA		-1.712	-2.065	-1.359	0.000	0.124	negative	carbon
SFo - MA		-1.689	-2.107	-1.272	0.000	0.147	negative	carbon
SFy - SFo		0.266	-0.123	0.656	0.385	0.137	no effect	carbon
UF - LF	**Dead wood pool**	0.463	-0.124	1.050	0.221	0.206	no effect	carbon
UF - LBF		0.278	-0.333	0.889	0.815	0.215	no effect	carbon
UF - PA		-1.125	-1.736	-0.514	0.000	0.215	negative	carbon
UF - MA		-1.107	-1.805	-0.408	0.000	0.246	negative	carbon
LF - LBF		-0.185	-0.556	0.186	0.740	0.130	no effect	carbon
LF - PA		-1.588	-1.964	-1.213	0.000	0.132	negative	carbon
LF - MA		-1.570	-2.060	-1.080	0.000	0.172	negative	carbon
LBF - PA		-1.403	-1.771	-1.036	0.000	0.129	negative	carbon
LBF - MA		-1.385	-1.870	-0.900	0.000	0.170	negative	carbon
PA - MA		0.018	-0.474	0.511	1.000	0.173	no effect	carbon
PA - SFy		0.464	0.031	0.897	0.027	0.152	positive	carbon
MA - SFy		0.445	-0.111	1.002	0.207	0.196	no effect	carbon
SFo - PA		-0.715	-1.205	-0.226	0.000	0.172	negative	carbon
SFo - MA		-0.697	-1.272	-0.122	0.007	0.202	negative	carbon
SFy - SFo		0.252	-0.287	0.790	0.796	0.189	no effect	carbon
UF - LF	**Soil pool**	0.314	-0.392	1.020	0.830	0.248	no effect	carbon
UF - LBF		0.366	-0.378	1.111	0.753	0.262	no effect	carbon
UF - PA		0.116	-0.628	0.860	0.999	0.262	no effect	carbon
UF - MA		0.065	-0.778	0.909	1.000	0.297	no effect	carbon
LF - LBF		0.052	-0.374	0.478	1.000	0.150	no effect	carbon
LF - PA		-0.198	-0.631	0.235	0.810	0.152	no effect	carbon
LF - MA		-0.249	-0.820	0.322	0.843	0.201	no effect	carbon
LBF - PA		-0.250	-0.672	0.171	0.558	0.148	no effect	carbon
LBF - MA		-0.301	-0.859	0.257	0.666	0.196	no effect	carbon
PA - MA		-0.051	-0.622	0.520	1.000	0.201	no effect	carbon
PA - SFy		0.073	-0.420	0.566	0.999	0.174	no effect	carbon
MA - SFy		0.124	-0.520	0.768	0.997	0.227	no effect	carbon
SFo - PA		-0.306	-0.865	0.253	0.652	0.197	no effect	carbon
SFo - MA		-0.356	-1.020	0.307	0.670	0.233	no effect	carbon
SFy - SFo		0.233	-0.378	0.843	0.910	0.215	no effect	carbon
UF - LF	**pH**	0.266	-0.265	0.797	0.737	0.187	no effect	soil
UF - LBF		0.701	0.138	1.264	0.005	0.198	positive	soil
UF - PA		1.763	1.201	2.325	0.000	0.198	positive	soil
UF - MA		1.493	0.859	2.127	0.000	0.223	positive	soil
LF - LBF		0.435	0.121	0.749	0.001	0.111	positive	soil
LF - PA		1.497	1.177	1.816	0.000	0.112	positive	soil
LF - MA		1.227	0.806	1.648	0.000	0.148	positive	soil
LBF - PA		1.062	0.751	1.372	0.000	0.109	positive	soil

LBF - MA		0.792	0.383	1.202	0.000	0.144	positive	soil
PA - MA		-0.270	-0.689	0.150	0.464	0.148	no effect	soil
PA - SFy		-0.764	-1.127	-0.402	0.000	0.128	negative	soil
MA - SFy		-0.495	-0.969	-0.021	0.034	0.167	negative	soil
SFo - PA		1.160	0.750	1.571	0.000	0.145	positive	soil
SFo - MA		0.891	0.404	1.378	0.000	0.172	positive	soil
SFy - SFo		-0.396	-0.844	0.052	0.120	0.158	no effect	soil
UF - LF	**N**	0.428	-0.126	0.983	0.243	0.195	no effect	soil
UF - LBF		0.572	-0.013	1.157	0.060	0.206	no effect	soil
UF - PA		0.177	-0.407	0.762	0.969	0.206	no effect	soil
UF - MA		0.092	-0.573	0.756	1.000	0.234	no effect	soil
LF - LBF		0.144	-0.194	0.481	0.858	0.119	no effect	soil
LF - PA		-0.251	-0.594	0.092	0.304	0.121	no effect	soil
LF - MA		-0.337	-0.789	0.115	0.284	0.159	no effect	soil
LBF - PA		-0.395	-0.729	-0.060	0.010	0.118	negative	soil
LBF - MA		-0.481	-0.923	-0.038	0.024	0.156	negative	soil
PA - MA		-0.086	-0.539	0.367	0.997	0.159	no effect	soil
PA - SFy		0.096	-0.295	0.487	0.990	0.138	no effect	soil
MA - SFy		0.181	-0.329	0.692	0.934	0.180	no effect	soil
SFo - PA		-0.412	-0.854	0.030	0.086	0.156	no effect	soil
SFo - MA		-0.498	-1.024	0.028	0.076	0.185	no effect	soil
SFy - SFo		0.316	-0.168	0.801	0.443	0.171	no effect	soil
UF - LF	**P**	0.430	-0.319	1.178	0.599	0.264	no effect	soil
UF - LBF		0.393	-0.401	1.187	0.748	0.279	no effect	soil
UF - PA		0.497	-0.295	1.290	0.492	0.279	no effect	soil
UF - MA		1.363	0.468	2.258	0.000	0.315	positive	soil
LF - LBF		-0.036	-0.482	0.409	1.000	0.157	no effect	soil
LF - PA		0.068	-0.385	0.520	0.999	0.159	no effect	soil
LF - MA		0.934	0.337	1.530	0.000	0.210	positive	soil
LBF - PA		0.104	-0.336	0.544	0.991	0.155	no effect	soil
LBF - MA		0.970	0.390	1.551	0.000	0.204	positive	soil
PA - MA		0.866	0.271	1.461	0.001	0.209	positive	soil
PA - SFy		-0.117	-0.630	0.396	0.993	0.181	no effect	soil
MA - SFy		-0.983	-1.655	-0.311	0.000	0.236	negative	soil
SFo - PA		0.376	-0.205	0.957	0.455	0.205	no effect	soil
SFo - MA		1.242	0.551	1.933	0.000	0.243	positive	soil
SFy - SFo		-0.259	-0.894	0.376	0.880	0.224	no effect	soil
UF - LF	**K**	0.170	-0.492	0.831	0.987	0.233	no effect	soil
UF - LBF		0.350	-0.338	1.038	0.724	0.242	no effect	soil
UF - PA		0.867	0.179	1.555	0.004	0.242	positive	soil
UF - MA		1.237	0.447	2.027	0.000	0.278	positive	soil
LF - LBF		0.180	-0.236	0.597	0.847	0.146	no effect	soil
LF - PA		0.698	0.276	1.120	0.000	0.148	positive	soil
LF - MA		1.067	0.510	1.625	0.000	0.196	positive	soil
LBF - PA		0.517	0.104	0.931	0.005	0.145	positive	soil
LBF - MA		0.887	0.335	1.439	0.000	0.194	positive	soil

PA - MA		0.369	-0.193	0.932	0.436	0.198	no effect	soil
PA - SFy		-0.567	-1.054	-0.080	0.012	0.171	negative	soil
MA - SFy		-0.936	-1.569	-0.303	0.000	0.223	negative	soil
SFo - PA		0.540	-0.013	1.093	0.060	0.194	no effect	soil
SFo - MA		0.909	0.255	1.564	0.001	0.230	positive	soil
SFy - SFo		0.027	-0.579	0.632	1.000	0.213	no effect	soil
UF - LF	**Na**	0.201	-0.424	0.826	0.959	0.220	no effect	soil
UF - LBF		0.211	-0.434	0.856	0.955	0.227	no effect	soil
UF - PA		0.433	-0.211	1.078	0.407	0.227	no effect	soil
UF - MA		0.425	-0.320	1.171	0.606	0.262	no effect	soil
LF - LBF		0.010	-0.392	0.412	1.000	0.141	no effect	soil
LF - PA		0.232	-0.174	0.639	0.604	0.143	no effect	soil
LF - MA		0.224	-0.313	0.762	0.869	0.189	no effect	soil
LBF - PA		0.222	-0.177	0.622	0.632	0.140	no effect	soil
LBF - MA		0.214	-0.322	0.751	0.890	0.189	no effect	soil
PA - MA		-0.008	-0.553	0.537	1.000	0.192	no effect	soil
PA - SFy		-0.452	-0.924	0.021	0.071	0.166	no effect	soil
MA - SFy		-0.443	-1.056	0.169	0.317	0.215	no effect	soil
SFo - PA		0.310	-0.226	0.847	0.591	0.189	no effect	soil
SFo - MA		0.302	-0.333	0.937	0.782	0.223	no effect	soil
SFy - SFo		0.141	-0.448	0.730	0.991	0.207	no effect	soil
UF - LF	**CaMg**	0.213	-0.434	0.861	0.954	0.228	no effect	soil
UF - LBF		0.875	0.192	1.557	0.004	0.240	positive	soil
UF - PA		1.403	0.721	2.085	0.000	0.240	positive	soil
UF - MA		1.572	0.798	2.346	0.000	0.272	positive	soil
LF - LBF		0.662	0.269	1.054	0.000	0.138	positive	soil
LF - PA		1.190	0.791	1.588	0.000	0.140	positive	soil
LF - MA		1.359	0.833	1.884	0.000	0.185	positive	soil
LBF - PA		0.528	0.140	0.916	0.001	0.137	positive	soil
LBF - MA		0.697	0.183	1.212	0.002	0.181	positive	soil
PA - MA		0.169	-0.357	0.695	0.959	0.185	no effect	soil
PA - SFy		-0.449	-0.903	0.006	0.055	0.160	no effect	soil
MA - SFy		-0.618	-1.211	-0.024	0.035	0.209	negative	soil
SFo - PA		0.720	0.205	1.235	0.001	0.181	positive	soil
SFo - MA		0.889	0.278	1.500	0.000	0.215	positive	soil
SFy - SFo		-0.271	-0.834	0.292	0.771	0.198	no effect	soil
UF - LF	**Al**	-0.052	-0.603	0.500	1.000	0.194	no effect	soil
UF - LBF		-0.376	-0.966	0.213	0.470	0.208	no effect	soil
UF - PA		-1.221	-1.808	-0.634	0.000	0.207	negative	soil
UF - MA		-1.373	-2.031	-0.714	0.000	0.232	negative	soil
LF - LBF		-0.325	-0.642	-0.008	0.041	0.112	negative	soil
LF - PA		-1.169	-1.492	-0.847	0.000	0.114	negative	soil
LF - MA		-1.321	-1.746	-0.896	0.000	0.150	negative	soil
LBF - PA		-0.845	-1.158	-0.532	0.000	0.110	negative	soil
LBF - MA		-0.996	-1.407	-0.586	0.000	0.144	negative	soil
PA - MA		-0.152	-0.573	0.270	0.930	0.148	no effect	soil

PA - SFy	0.382	0.019	0.745	0.032	0.128	positive	soil
MA - SFy	0.534	0.057	1.010	0.017	0.168	positive	soil
SFo - PA	-0.891	-1.302	-0.479	0.000	0.145	negative	soil
SFo - MA	-1.042	-1.531	-0.553	0.000	0.172	negative	soil
SFy - SFo	0.508	0.060	0.957	0.015	0.158	positive	soil

Table S7. Effect sizes, the lower and upper confidence interval (CI) and the p-value obtained by the approach of Linear Mixed Effect Models (LMM) and Quantile Generalized Additive Models (qGAM) for the land-use and land-cover transition (LULC) that showed changes in the significance when comparing both approaches in the validation analysis. Small trees = DAP < 10 cm, large trees = DAP ≥ 10 cm. UF: Undisturbed primary forest, LF: Logged primary forest, LBF: Logged-and-burned primary forest, SFo: Old secondary forest, SFy: Young secondary forest, PA: Pasture, MA: Mechanized agriculture.

Variable	LULC transition	ES LMM	Lwr LMM	Upr LMM	P value LMM	ES qGAM	Lwr qGAM	Upr qGAM	P value qGAM	Change in significance (LMM to qGAM)
Ant richness	SFo - PA	-0.535	0.021	-1.090	0.068	-0.593	-0.101	-1.086	0.008	Became significant
Liana richness	LF - LBF	-0.367	-0.054	-0.679	0.011	-0.296	0.020	-0.613	0.082	Became non-significant
Small tree richness	UF - LBF	-0.121	0.199	-0.441	0.913	-0.277	-0.029	-0.525	0.018	Became significant
Orchid bee richness	LF - MA	-0.828	0.035	-1.691	0.069	-0.776	-0.002	-1.551	0.050	Became significant
Orchid bee richness	LBF - MA	-0.766	0.039	-1.571	0.073	-0.713	-0.017	-1.410	0.041	Became significant
Orchid bee richness	SFy - SFo	-1.083	0.031	-2.196	0.062	-1.141	-0.140	-2.141	0.015	Became significant
Dead wood carbon pool	PA - SFy	0.464	0.897	0.031	0.027	0.193	0.405	-0.018	0.098	Became non-significant
Soil pH	SFy - SFo	-0.396	0.052	-0.844	0.120	-0.412	-0.020	-0.805	0.033	Became significant
Soil N	SFo - MA	-0.498	0.029	-1.024	0.076	-0.442	-0.046	-0.838	0.018	Became significant
Soil K	PA - MA	0.369	0.932	-0.193	0.435	0.616	1.054	0.178	0.001	Became significant
Soil Na	PA - SFy	-0.452	0.021	-0.924	0.071	-0.313	-0.015	-0.612	0.033	Became significant
Soil Na	MA_SFy	-0.443	0.169	-1.056	0.317	-0.490	-0.064	-0.916	0.013	Became significant

SI References

1. A. Zanne, *et al.*, Data from: Towards a worldwide wood economics spectrum. *Dryad Digit. Repos.* (2009) https:/doi.org/https://doi.org/10.5061/dryad.234.
2. J. Chave, *et al.*, Tree allometry and improved estimation of carbon stocks and balance in tropical forests. *Oecologia* **145**, 87–99 (2005).
3. B. W. Nelson, *et al.*, Allometric regressions for improved estimate of secondary forest biomass in the central Amazon. *For. Ecol. Manage.* **117**, 149–167 (1999).
4. J. J. Gerwing, L. Farias, Integrating liana abundance and forest stature into an estimate of total aboveground biomass for an eastern Amazonian forest. *J. Trop. Ecol.* **16**, 327–335 (2000).
5. T. A. Gardner, *et al.*, A social and ecological assessment of tropical land uses at multiple scales: the Sustainable Amazon Network. *Philos. Trans. R. Soc. B Biol. Sci.* **368**, 20120166 (2013).
6. E. Berenguer, *et al.*, A large-scale field assessment of carbon stocks in human-modified tropical forests. *Glob. Chang. Biol.* **20**, 3713–3726 (2014).
7. A. C. Lees, *et al.*, One hundred and thirty-five years of avifaunal surveys around Santarém, central Brazilian Amazon. *Rev. Bras. Ornitol.* **21**, 16–57 (2013).
8. A. C. Lees, *et al.*, Paragominas: A quantitative baseline inventory of an eastern Amazonian avifauna. *Rev. Bras. Ornitol.* **20**, 93–118 (2012).
9. R. R. de C. Solar, J. C. M. Chaul, M. Maués, J. H. Schoereder, A Quantitative Baseline of Ants and Orchid Bees in Human-Modified Amazonian Landscapes in Paragominas, PA, Brazil. *Sociobiology* **63**, 925 (2016).
10. R. C. Team, R: A language and environment for statistical computing (2021).
11. M. Fasiolo, S. N. Wood, M. Zaffran, R. Nedellec, Y. Goude, qgam : Bayesian Nonparametric Quantile Regression Modeling in R. *J. Stat. Softw.* **100**, 1–31 (2021).
12. T. Hothorn, F. Bretz, P. Westfall, Simultaneous inference in general parametric models. *Biometrical J.* **50**, 346–363 (2008).
13. C. M. Souza, *et al.*, Reconstructing Three Decades of Land Use and Land Cover Changes in Brazilian Biomes with Landsat Archive and Earth Engine. *Remote Sens.* **12**, 2735 (2020).
14. E. A. T. Matricardi, *et al.*, Long-term forest degradation surpasses deforestation in the Brazilian Amazon. *Science (80-.).* **369**, 1378–1382 (2020).
15. F. Hartig, DHARMa: Residual Diagnostics for Hierarchical (Multi-Level / Mixed) Regression Models. R package version 0.4.5. (2022).
